# Extracts from *Eleutherococcus senticosus* (Rupr. et Maxim.) Maxim. Roots: A New Hope Against Honeybee Death Caused by Nosemosis

**DOI:** 10.3390/molecules25194452

**Published:** 2020-09-28

**Authors:** Aneta A. Ptaszyńska, Daniel Załuski

**Affiliations:** 1 Department of Immunobiology, Institute of Biological Sciences, Faculty of Biology and Biotechnology, Maria Curie-Skłodowska University, Akademicka 19 Str., 20-033 Lublin, Poland; 2 Department of Pharmaceutical Botany and Pharmacognosy, Ludwik Rydygier Collegium Medicum, Nicolaus Copernicus University, Marie Curie-Skłodowska 9, 85-094 Bydgoszcz, Poland; daniel_zaluski@onet.eu

**Keywords:** honeybee, *Apis mellifera*, *Eleutherococcus senticosus*, *Garcinia cambogia*, *Panax ginseng*, *Ginkgo biloba*, *Schisandra chinensis*, *Camellia sinensis*, fumagillin, eleutherosides, adaptogenic herbs, insectageddon

## Abstract

Pollinators, the cornerstones of our terrestrial ecosystem, have been at the very core of our anxiety. This is because we can nowadays observe a dangerous decline in the number of insects. With the numbers of pollinators dramatically declining worldwide, the scientific community has been growing more and more concerned about the future of insects as fundamental elements of most terrestrial ecosystems. Trying to address this issue, we looked for substances that might increase bee resistance. To this end, we checked the effects of plant-based adaptogens on honeybees in laboratory tests and during field studies on 30 honeybee colonies during two seasons. In this study, we have tested extracts obtained from: *Eleutherococcus senticosus, Garcinia cambogia, Panax ginseng, Ginkgo biloba, Schisandra chinensis*, and *Camellia sinensis*. The 75% ethanol *E. senticosus* root extract proved to be the most effective, both as a cure and in the prophylaxis of nosemosis. Therefore, *Eleutherococcus senticosus*, and its active compounds, eleutherosides, are considered the most powerful adaptogens, in the pool of all extracts that were selected for screening, for supporting immunity and improving resistance of honeybees. The optimum effective concentration of 0.4 mg/mL *E. senticosus* extract responded to c.a. 5.76, 2.56 and 0.07 µg/mL of eleutheroside B, eleutheroside E and naringenin, respectively. The effect of *E. senticosus* extracts on honeybees involved a similar adaptogenic response as on other animals, including humans. In this research, we show for the first time such an adaptogenic impact on invertebrates, i.e., the effect on honeybees stressed by nosemosis. We additionally hypothesised that these adaptogenic properties were connected with eleutherosides—secondary metabolites found exclusively in the *Eleutherococcus* genus and undetected in other studied extracts. As was indicated in this study, eleutherosides are very stable chemically and can be found in extracts in similar amounts even after two years from extraction. Considering the role bees play in nature, we may conclude that demonstrating the adaptogenic properties which plant extracts have in insects is the most significant finding resulting from this research. This knowledge might bring to fruition numerous economic and ecological benefits.

## 1. Introduction

The very existence of humans on the planet depends on other organisms’ well-being, also on insects—especially the main managed pollinator—the honeybee [1,2]. We are now living in the Anthropocene—a geological epoch characterised by the commencement of a significant human impact on the Earth’s geology and ecosystems [3,4,5]. It is marked by the sixth mass extinction [6]. Insects suffer an extinction rate that is up to 8 times higher than in other groups of most endangered species [7]. In Germany, during the routine monitoring of protected areas, scientists found that within 27 years of researching the same sites, insect biomass decreased by 76%, and in the middle of the summer, the decline in flying insect biomass was up to 82% [8]. Similar trends have been observed around the world. Sánchez-Bayo and Wyckhuys [9] compared 73 long-term insect tests in their review and showed that the rate of biodiversity loss in this group of animals is so large that 40% of insect species in the world may die over the next few years. Due to its enormous impact on the environment, this decrease in the number and biodiversity of insect populations is sometimes called the insectageddon. This rapid insect extinction is connected with malnutrition, enormous pesticide overuse and diseases attacking weakened insects. Nosemosis is one of such diseases attacking weakened honeybees. It is caused by microsporidia from the *Nosema* genus. Until recently, only two types of *Nosema* species were thought to parasitize bees, i.e., *Nosema apis* and *N*. *ceranae* [10,11], however, the third genus, *N*. *neumanni,* was described in 2017 [12]. Nosemosis negatively alters the gut epithelium renewal rate [13] and creates a layer of mature spores on the intestine surface [14], which leads to bee malnutrition and changes in the composition of microelements [15]. Nosemosis also changed honeybee intestinal microbiome contents, both on bacterial [16] and yeast [17] levels. Furthermore, it causes impairments of many gland functions [18,19,20], accelerates age polyethism in young honeybees [19], decreases the level of hormone secretion and causes anatomical changes in bee queens’ ovaries, and finally, it affects fertility and the survival rate in older honeybee males [21,22,23]. All these factors affect both individual honeybees and the whole bee colony, decreasing its survival rate [13,14,15,16,17,18,19,20,21,22,23].

Therefore, various actions that increase pollinators’ wellbeing are important, such as creating flower strips, pollinator-friendly gardens, reducing pesticide applications and raising public awareness of pollinators’ importance. It is also essential to look for various ways to enhance the immunity of pollinators in order to help them survive. Some of the substances increasing immunity can be single plant-derived secondary metabolites, which when transported into nectar, can be beneficial for pollinators [24]. Other agents that might look like effective ‘bee medicines’ can be natural products such as plant extracts, essential oils and phytochemicals [25]. The following extracts have been tested to date, albeit without much success: *Achillea alpina* (Ledeb), *Achyranthes japonica* (Miq.), *Allium senescens* L. var. *senescens*, *Amaranthus mangostanys* L., *Aster tataricus* L.f., *Astilboides tabularis* (Hemsl.) Engl., *Astragalus membranaceus* Bunge var. *membranaceus*, *Cinnamomum cassia* (L.) J. Presl, *Cirsium nipponicum* (Maxim.) Makino, *Cryptocarya alba* (Molina) Looser, *Cyrtomium fortune* J. Sm., *Disporum uniflorum* Baker, *Eucalyptus citridora* (Hook.) K.D. Hill, L.A.S.Johnson, *Lythrum salicaria* L., *Mentha arvensis* L., *Perilla frutescens* var. *acuta* Kudo, *Physalis alkekengi* var. *francheti* (Mast.) Hort, *Rheum undulatum* L., *Symphytum officinale* L. and *Veratrum oxysepalum* Turcz. [26,27]. It is worth emphasising that only extracts from two representatives of the *Compositae* family, *Artemisia dubia* (Wall.) and *Aster scaber* Thunberg, at non-toxic concentrations, proved to show anti-nosemosis effects [28,29]. In several studies, essential oils such as mint (*Mentha pepper* L.), melissa (*Melissa officinalis* L.), coriandrum (*Coriander sativum* L.), thyme (*Satureja hortensis* L.) oils, other phytochemicals such as caffeine, clove oil, gallic acid, kaempferol and p-coumaric acid, and even propolis expanded honeybee longevity and to some extent, reduced nosemosis levels [27,28,29,30,31,32,33,34,35]. Such phytochemicals as clove oil, laurel extract and sulforaphane, moderately decreased *Nosema*-infection, but at the same time, strongly reduced honeybee lifespan [33,36,37]. Other tested phytochemicals, such as amygdalin, anabasine, aucubin, catalpol, nicotine and thymol, had no effect against *Nosema* infection [33,36,37].

Nonetheless, new ‘bee medicines’ should be searched for, especially now when fumagillin is no longer effective in the treatment of nosemosis as *N. ceranae* might evade it [38,39], and because its use is prohibited in EU countries (A maximum residue level, MRL; Commission Regulation, EU, 2010, no. 37/2010). The use of medicines in apiculture should be safe both for honeybees and for humans. Fumagillin is the cause of alterations to the ultrastructure of hypopharyngeal glands in bees and causes chromosomal aberrations, and carcinogenicity in humans [40]. Therefore, there is a significant demand for a new medication that safely and effectively treats honeybee colonies infected with *N. ceranae*.

Looking for substances that would increase bee resistance, we checked the effects of plant-based adaptogens, which are defined as natural compounds or plant extracts that increase the adaptability, resilience and survival of living organisms subjected to stress [41]. This study, being an element of a program searching for bioactive constituents, was aimed at determining whether adaptogenic plants (*Eleutherococcus senticosus* (Rupr. et Maxim.) Maxim. and Oliv, *Eleutherococcus henryi* Oliv., *Garcinia gummi-gutta* (L.) N. Robson, *Panax ginseng* C.A. Meyer, *Schisandra chinensis* (Turcz.) Baill., *Camellia sinensis* (L.) Kuntze and *Ginkgo biloba* L.) might be effective and safe candidates for the treatment and prophylaxis of nosemosis. 

## 2. Results

We tested six adaptogenic plant extracts, i.e., *Eleutherococcus senticosus*, *Garcinia cambogia*, *Panax ginseng*, *Ginkgo biloba*, *Schisandra chinensis* and *Camellia sinensis*. The 75% ethanol *E. senticosus* root extract rich in eleutherosides proved to be most effective, both as a cure and in the prophylaxis of nosemosis. The minimum effective dose was established at 0.4 mg/mL of *E. senticosus* extract. It proved to be optimal for honeybee nosemosis treatment and prophylaxis in laboratory studies and field tests conducted on 30 honeybee colonies trough two seasons. 

### 2.1. Results of Cage Tests

Emerging honeybees were divided randomly into three treatment variants, “A”, “B”, and “C” (all acronyms are defined in Supplementary Materials Tables S1–S3):

A. Honeybees in variant “A” (control, impact of extracts on uninfected honeybees) served as a control and were not infected with *N. ceranae*. Feeding group SS (sucrose syrup) served as a negative control with uninfected honeybees fed solely with 1:1 w:v [1:1 weight:volume sugar:water] sucrose solution and not treated with extracts.
B. In variant “B” (impact of extracts on the treatment of nosemosis), to check whether the supplementation of honeybee diets with extracts influences the course of nosemosis, firstly, honeybees were *Nosema ceranae*-infected, and subsequently fed with a sucrose solution, containing extracts. Feeding group SS served as a negative control with *Nosema ceranae*-infected honeybees fed solely with a 1:1 w:v sucrose solution, not treated with extracts, and Fum (fumagillin), served as a positive control in the treatment of nosemosis.
C. In variant “C” (impact of extracts on the prevention of nosemosis), to check whether the supplementation of honeybee diets with extracts protects honeybees against nosemosis, firstly, honeybees were fed with a sucrose solution supplemented with extracts, and after that, were infected with *N. ceranae* spores. Feeding group SS served as a negative control with *Nosema ceranae*-infected honeybees fed solely with a 1:1 w:v sucrose solution and not treated with extracts.

#### 2.1.1. Results of Cage Tests: Screening of Commercial Plant Extracts

**Figure 1:**
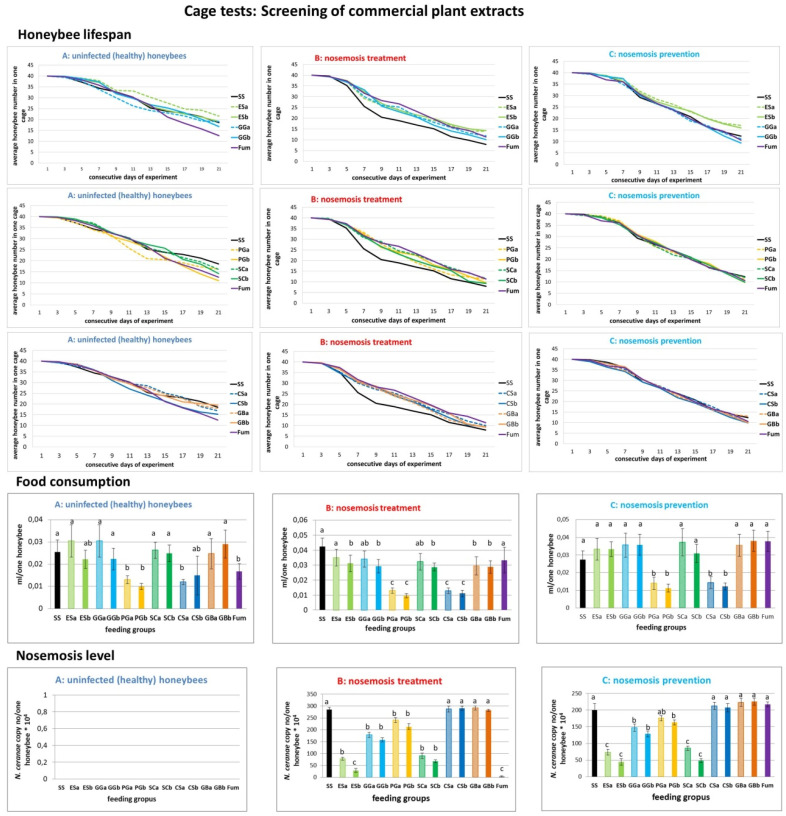
Results of cage tests: Screening of commercial plant extracts. Data illustrated honeybee lifespan, food consumption and nosemosis level in all three variants of experiments conducted, i.e., (**A**) uninfected honeybees, (**B**) treatment of nosemosis and (**C**) prevention of nosemosis were compared. Lowercase letters (a,b,c) indicate significant differences between the group fed pure sucrose syrup without extracts (SS) and the groups fed with additions of extracts (at least *p* ≤ 0.005 for the honeybee lifespan and nosemosis level, and *p* ≤ 0.05 for food consumption) (analysis of variance (ANOVA), Tukey test). Error bars denote a confidence interval (CI). Feeding groups (acronyms are defined in Supplementary Materials Table S1): (SS—sucrose syrup) control, pure sucrose syrup without extracts, (ESa) sucrose syrup supplemented with 0.2 mg/mL of commercial extracts of *E. senticosus*, (ESb) sucrose syrup supplemented with 1 mg/mL of commercial extracts of *E. senticosus*, (GGa) sucrose syrup supplemented with 0.2 mg/mL of commercial extracts of *G. gummi-gutta*, (GGb) sucrose syrup supplemented with 1 mg/mL of commercial extracts of *G. gummi-gutta*, (PGa) sucrose syrup supplemented with 0.2 mg/mL of commercial extracts of *P. ginseng*, (PGb) sucrose syrup supplemented with 1 mg/mL of commercial extracts of *P. ginseng*, (SCa) sucrose syrup supplemented with 0.2 mg/mL of commercial extracts of *S. chinensis*, (SCb) sucrose syrup supplemented with 1 mg/mL of commercial extracts of *S. chinensis*, (CSa) sucrose syrup supplemented with 0.2 mg/mL of commercial extracts of *C. sinensis*, (CSb) sucrose syrup supplemented with 1 mg/mL of commercial extracts of *C. sinensis*, (GBa) sucrose syrup supplemented with 0.2 mg/mL of commercial extracts of *G. biloba*, (GBb) sucrose syrup supplemented with 1 mg/mL of commercial extracts of *G. biloba*, and (Fum) fumagillin as a positive control in the treatment of nosemosis.

We compared data from all three treatment methods (A, B, and C, acronyms are defined in Supplementary Materials Table S1) of conducted experiments (Table 1, Figure 1), i.e., A (lifespan), B (*Nosema*-treatment), C (*Nosema*-prevention), to choose the best extract for further experiments. In variant B, honeybee lifetime extension (with *p* < 0.05) was observed after feeding with all tested plant extracts. However, not all extracts reduced the level of nosemosis. This may have been caused by better nutrition of infected honeybees that were fed with additional extracts compared to the control group fed only with the sucrose solution.

Commercial extracts from *Eleutherococcus senticosus* (groups: ESa and Esb, Figure 1) had the best impact on honeybees out of all the studied commercial extracts. Honeybees were fed with extracts in concentrations of 0.2 and 1 mg of active compound in 1 mL of the sucrose solution. The 1 mg/mL *E. senticossus* extract had the most powerful impact on the level of nosemosis, but the concentration of 0.2 mg/mL was more eagerly consumed by honeybees and more efficiently prolonged honeybee lifespans than 1 mg/mL (groups: ESa and Esb, Figure 1). Therefore, we chose *E. senticosus* extracts for further experiments and decided to check the dose higher than 0.2 mg/mL, i.e., 0.4 mg/mL.

Commercial extract from *Garcinia gummi-gutta* (groups: GGa and GGb, Figure 1) was consumed in similar amounts as in the control group (SS) fed with the pure sucrose solution. Mortality was also similar to the control group (SS) in all three (A, B, C) experiment variants. Additionally, *G. gummi-gutta* extract reduced the level of nosemosis both in the variant B (course) and C (prevention).

Commercial extract from *Panax ginseng* (groups: PGa and PGb, Figure 1) was only reluctantly consumed by honeybees. In variant A, *P. ginseng* in the concentration of 1 mg/mL (group PGb) increased mortality of honeybees. In variants B and C, the mortality was similar to the mortality observed in the control group (SS). Both in variant B (course) and C (prevention), *P. ginseng* extract slightly reduced the nosemosis level in comparison to the control (SS).

Commercial extract from *Schisandra chinensis* (groups: SCa and SCb, Figure 1) was consumed in a similar amount as in the control group (SS) fed with the pure sugar syrup. Mortality was also similar to the control group (SS) in all three (A, B, C) experiment variants. Additionally, *S. chinensis* extract reduced the nosemosis level both in variant B (course) and C (prevention).

Commercial extract from *Camellia sinensis* (groups: CSa and CSb, Figure 1) was only reluctantly consumed by honeybees. In variant A, *C. sinensis* in the concentration of 1 mg/mL (group CSb) increased mortality of honeybees. In variants B and C, the mortality was similar to the mortality observed for the control group (SS). Additionally, *C. sinensis* did not reduce the nosemosis level in comparison to the control (SS) both in variant B (course) and C (prevention).

Commercial extract from *Ginkgo biloba* (groups: GBa and GBb, Figure 1) was consumed in a similar amount as in the control group (SS) fed with the pure sucrose solution. Mortality was also similar to the control group (SS) in all three (A, B, C) experiment variants. *G. biloba* did not reduce the level of nosemosis in comparison to the control (SS) neither in variant B (course) nor in C (prevention).

Fumagillin (Fum, Figure 1) was consumed in a slightly smaller amount in variant A and in a similar amount in the control group (SS) fed with the pure sucrose solution in other variants. In variant A, fumagillin increased mortality of honeybees. In variants B and C, mortality was similar to the mortality observed for the control group (SS). In variant B (nosemosis treatment), fumagillin strongly reduced the level of nosemosis but it was ineffective in the prevention of nosemosis (variant C).

#### 2.1.2. Results of Cage Tests: Screening for the Best Method to Obtain the *Eleutherococcus* Extract

**Figure 2:**
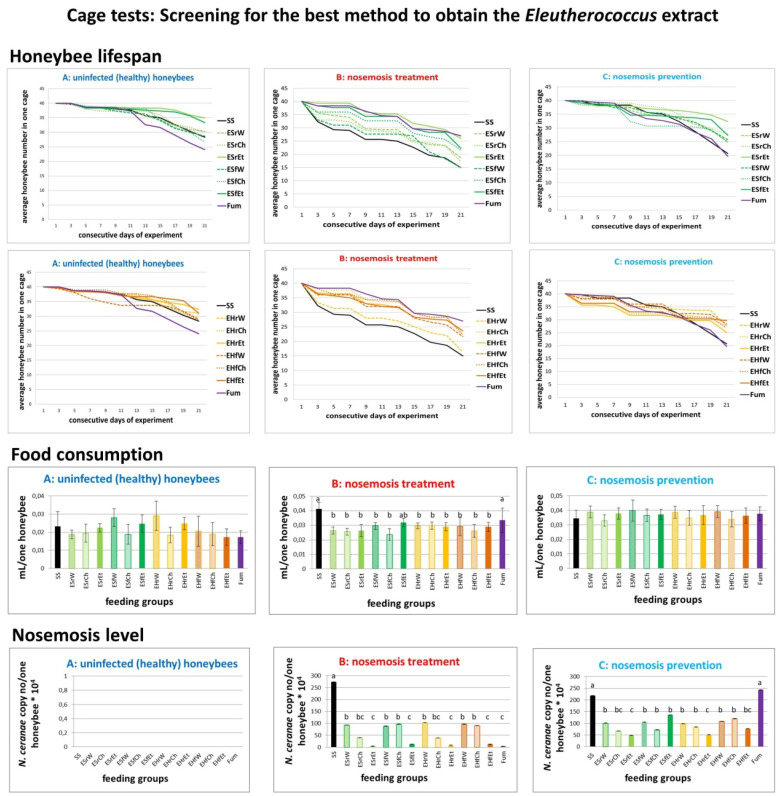
Results of cage tests: Screening for the best method of obtaining the *Eleutherococcus* extract. Data illustrated honeybee lifespan, food consumption and levels of nosemosis in all three variants of experiments conducted, i.e., (**A**) uninfected honeybees, (**B**) treatment of nosemosis and (**C**) prevention of nosemosis were compared. Lowercase letters (a,b,c) indicate significant differences between the group fed pure sucrose syrup without extracts (SS) and the groups fed with additions of extracts (at least *p* ≤ 0.05 for the honeybee lifespan, *p* ≤ 0.01 for food consumption and *p* ≤ 0.005 for level of nosemosis) (ANOVA, Tukey test). Error bars denote a confidence interval (CI). Laboratory extracts of *E. senticosus* (ES) and *E. henryi* (EH) were obtained using three different methods (water, W, chloroform, Ch, and ethanol, Et, extraction) from these plants’ roots and fruits (Table 2). 14 feeding groups (acronyms are defined in Supplementary Materials Table S2), i.e., (1) SS (control, pure sucrose syrup), (2) ESrW, (3) ESrCh, (4) ESrEt, (5) ESfW, (6) ESfCh, (7) ESfEt, (8) EHrW, (9) EHrCh, (10) EHrEt, (11) EHfW, (12) EHfCh, (13) EHfEt, (14) Fum (positive control in the treatment of nosemosis, fumagillin). All pure laboratory extracts were added in the concentration of 0.4 mg/mL to compare three different methods of obtaining extracts.

Laboratory extracts of *E. senticosus* (ES) and *E. henryi* (EH) were obtained using three different methods of extraction, i.e., water, chloroform and ethanol from these plants’ roots and fruits (Table 2, acronyms are defined in Supplementary Materials Table S2). *E. senticossus* root ethanol extract prolonged bee lifespan, strongly reduced level of nosemosis and in variant A, was consumed at a comparable level to the control group fed only the sucrose syrup (Figure 2). Generally, *E. senticossus* root ethanol extract was the most effective in prophylaxis and in the treatment of nosemosis. Therefore, we chose the *E. senticosus* root ethanol extract for further experiments.

#### 2.1.3. Results of Cage Tests: Screening for the Best *Eleutherococcus* Dose

**Figure 3:**
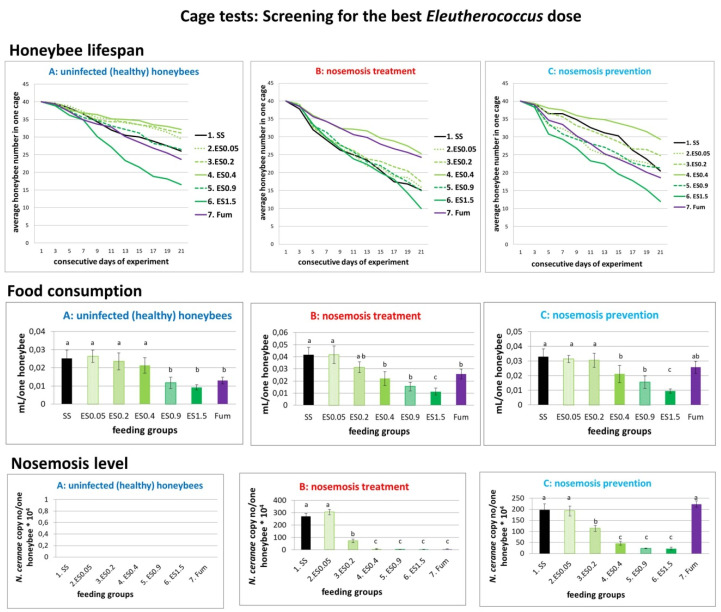
Results of cage tests: Screening for the best *Eleutherococcus* dose. Data illustrated honeybee lifespan, food consumption and the level of nosemosis in all three variants of conducted experiments, i.e., (**A**) uninfected honeybees, (**B**) treatment of nosemosis and (**C**) prevention of nosemosis were compared. Lowercase letters (a,b,c) indicate significant differences between the group fed with pure sucrose syrup without extracts (SS) and the groups fed with additions of extracts (at least *p* ≤ 0.05 for the honeybee lifespan, *p* ≤ 0.05 for food consumption and *p* ≤ 0.01 for level of nosemosis) (ANOVA, Tukey test). Error bars denote a confidence interval (CI). Feeding groups: *E. senticosus* ethanol root extract was added to the sucrose solution to the final concentrations of 0.05 mg/mL, 0.2 mg/mL, 0.4 mg/mL, 0.9 mg/mL and 1.5 mg/mL. Uninfected and *Nosema*-infected honeybees from A, B and C variants were divided into 14 feeding groups (acronyms are defined in Supplementary Materials Table S3): (1. SS) control, pure sucrose syrup without extracts, (2. ES0.05) sucrose syrup supplemented with 0.05 mg/mL *E. senticosus* extract, (3. ES0.2) sucrose syrup supplemented with 0.2 mg/mL *E. senticosus* extract, (4. ES0.4) sucrose syrup supplemented with 0.4 mg/mL *E. senticosus* extract, (5. ES0.9) sucrose syrup supplemented with 0.09 mg/mL *E. senticosus* extract, (6. ES1.5) sucrose syrup supplemented with 1.5 mg/mL *E. senticosus* extract, (7. Fum) fumagillin, positive control in the treatment of nosemosis.

Laboratory *E. senticosus* ethanol root extract was added to the sucrose solution to the final concentrations of 0.05 mg/mL, 0.2 mg/mL, 0.4 mg/mL, 0.9 mg/mL and 1.5 mg/mL (Figure 3, acronyms are defined in Supplementary Materials Table S3). *E. senticossus* in 0.4 mg/mL concentration visibly prolonged bee lifespan, strongly reduced the level of nosemosis and was eagerly consumed in variant A. Although *E. senticossus* in 1.5 mg/mL concentration reduced the level of nosemosis, it also decreased honeybee lifespan, and was very reluctantly consumed. Increased mortality in the 1.5 mg/mL group could probably be associated with the extract’s toxicity for honeybees. Further studies on pure substances should clarify this question. According to data from Supplementary Materials Table S4, the selected most effective concentration of 0.4 mg/mL *E. senticosus* extract responded to 5.76 µg/mL of eleutheroside B, 2.56 µg/mL of eleutheroside E and 0.068 µg/mL of naringenin.

Taking into account that the average syrup consumption for 0.4 mg/mL *E. senticosus* extract supplementation was 0.021395 (±0.006838), 0.022005 (±0.009454) and 0.021217 (±0.009464) mL/honeybee per day in the groups A, B and C respectively (Figure 3), the average sucrose solution consumption was equal to 0.02154 (±0.008585) mL/honeybee per day. Therefore, during 6 days of extract supplementation, each honeybee received c.a. 0.744 µg of eleutheroside B, 0.331 µg of eleutheroside E and 0.009 µg of naringenin. The optimum dose calculated per one honeybee/day was 0.124 µg of eleutheroside B, 0.055 µg of eleutheroside E and 0.001 µg of naringenin.

The toxic effect observed for the 1.5 mg/mL *E. senticosus* extract responded to 21.6 µg/mL of eleutheroside B, 9.6 µg/mL of eleutheroside E and 0.255 µg/mL of naringenin. The toxic effect of 1.5 mg/mL *E. senticosus* extract was clearly observed in the groups A (control, uninfected honeybees) and C (prevention of nosemosis), whereas in the group B (treatment of nosemosis), it was probably firstly covered by the curing effect of the extract treatment on the course of nosemosis (Figure 3). Nevertheless, the toxic effects of eleutherosides should be checked in further studies conducted on pure substances. Furthermore, other compounds also present in these plant extracts, such as, e.g., piperasines, chiisanoside or sessilines, should be checked as well, because their effects on honeybees are unknown.

Taking into account that the average syrup consumption for 1.5 mg/mL *E. senticosus* extract supplementation was 0.00927 (±0.002259), 0.01128 (±0.004732) and 0.00951 (±0.002207) mL/honeybee per day in groups A, B and C respectively (Figure 3), the average sucrose solution consumption was equal to 0.01002 (± 0.003066) mL/honeybee per day. Therefore, during 6 days of extract supplementation, each honeybee received c.a. 1.299 µg of eleutheroside B, 0.577 µg of eleutheroside E and 0.015 µg of naringenin. The toxic dose calculated per one honeybee/day was 0.216 µg of eleutheroside B, 0.096 µg of eleutheroside E and 0.003 µg of naringenin. Nevertheless, the toxic effects of eleutherosides should be checked in further studies conducted on pure substances.

Summarising, 0.4 mg/mL *E. senticosus* extract concentration was most beneficial in cage tests. Therefore, we chose this concentration for field apiary tests. However, in their natural environment, honeybees are not only fed supplements provided by beekeepers, but also forage on their natural floral resources. Consequently, we also picked higher *E. senticosus* extract concentration of 0.9 mg/mL for field studies, which was safe for honeybees and effective in the treatment of nosemosis during cage tests.

### 2.2. Additional Tests

#### 2.2.1. Results of Field Tests

15 healthy colonies with similar strength and 15 medium infected colonies with the similar infestation level between 140.77 × 10^4^ (±24.86) and 166.4 × 10^4^ (±23.94) *Nosema* copy number/bee were selected for the experiment. Differences among selected colonies were not significant (*p* > 0.05). Colonies in each apiary were divided into 6 groups, i.e., (F1) 5 colonies as control, an untreated group with healthy honeybees, (F2) 5 colonies with healthy honeybees fed with *E. senticosus* extract in the concentration of 0.4 mg/mL, (F3) 5 colonies with healthy honeybees fed with *E. senticosus* extract in the concentration of 0.9 mg/mL, (F4) 5 colonies with the control, untreated group with *Nosema*-infected honeybees, (F5) 5 colonies with *Nosema*-infected honeybees fed with *E. senticosus* extract in the concentration of 0.4 mg/mL and (F6) 5 colonies with *Nosema*-infected honeybees fed with *E. senticosus* extract in the concentration of 0.9 mg/mL.

**Figure 4:**
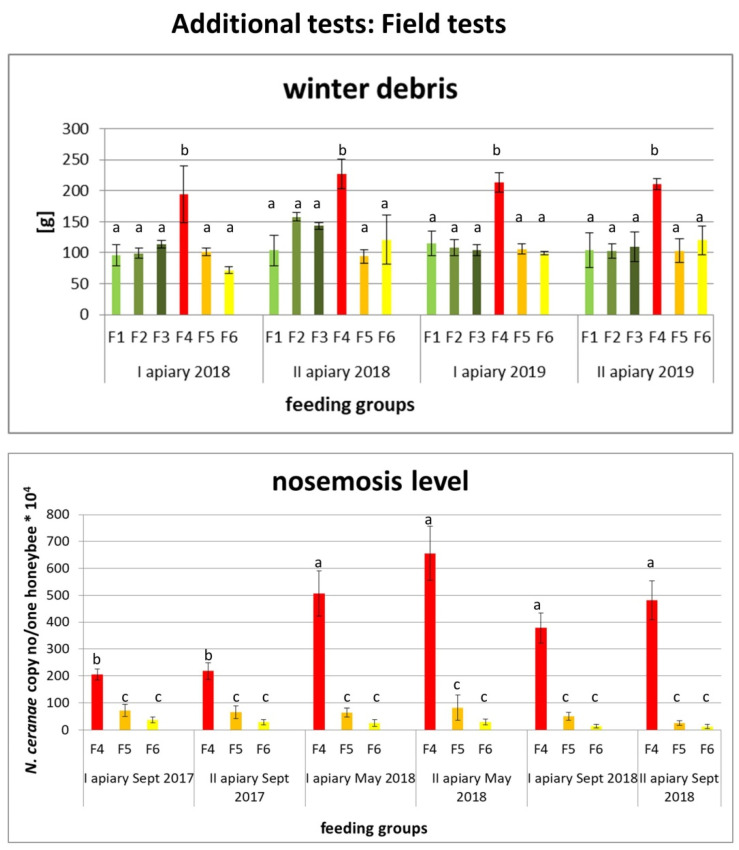
Results of additional tests: Field tests. Data illustrated winter debris and average levels of nosemosis in honeybee colonies fed with pure sucrose solution without extraxts (F1 and F4 groups with uninfected and *Nosema*-infected colonies, respectively), with *E. senticosus* extract in the concentration of 0.4 mg/mL (F2 and F5 groups with uninfected and *Nosema*-infected colonies, respectively) and with *E. senticosus* extract in the concentration of 0.9 mg/mL (F3 and F6 groups with uninfected and *Nosema*-infected colonies, respectively). Error bars denote a confidence interval (CI). In each apiary, colonies were divided into 6 groups, i.e., (F1) 5 colonies as control, untreated group with healthy honeybees, (F2) 5 colonies with healthy honeybees fed with *E. senticosus* extract in the concentration of 0.4 mg/mL, (F3) 5 colonies with healthy honeybees fed with *E. senticosus* extract in the concentration of 0.9 mg/mL, (F4) 5 untreated colonies with *Nosema*-infected honeybees, (F5) 5 colonies with *Nosema*-infected honeybees fed with *E. senticosus* extract in the concentration of 0.4 mg/mL and (F6) 5 colonies with *Nosema*-infected honeybees fed with *E. senticosus* extract in the concentration of 0.9 mg/mL.

Honeybee winter debris weight was similar in 2018 in all experimental colonies, but in 2019, there were significant differences; in the F4 group (*Nosema*-infected honeybees untreated with *E. senticosus* extract), winter debris was even two times higher than in other groups of infected honeybees fed with *E. senticosus* extracts. After the first season of treating honeybee colonies with *E. senticosus* extract, the level of nosemosis in treated groups was strongly reduced, and in colonies from F5–F6 groups fed with *E. senticosus* extracts, it was even 57 times lower than in the control group with *Nosema*-infected honeybees untreated with *E. senticosus* extract. The *E. senticosus* extract in the concentration of 0.9 mg/mL was more effective during the treatment of nosemosis in the first season (*p* < 0.01). During the second treatment season, the differences between colonies treated with 0.4 and 0.9 mg/mL extract concentrations blurred and were not relevant (*p* > 0.5); at the same time, there were no differences between these groups in weight of winter debris (Figure 4).

#### 2.2.2. Results of LIVE/DEAD Tests and *Nosema* Spore Cell Wall Analysis under Scanning Electron Microscopy (SEM)

The pre-treatment of *N. ceranae* spores with *Eleutherococcus* extracts had no effect on spores’ viability, their cell wall sculpture (Supplementary Figure S1) and furthermore, their ability to infect honeybees.

The pre-treatment of *N. ceranae* spores with *E. senticosus* in concentrations 0.4 and 0.9 mg/mL had no impact on spores’ viability observed after using dyes: SYTO 9 and propidium iodide, nor the ability to infect honeybees (n = 300; *p* > 0.5, analysis of variance (ANOVA) and Tukey test). The number of live spores in the control was 67.56 × 10^4^ (±3.973), after pre-treatment with *E. senticosus* in concentrations of 0.4 mg/mL, it was 66.81 × 10^4^ (±5.355) and after pre-treatment with *E. senticosus* in concentrations of 0.9 mg/mL, it was 67.14 × 10^4^ (±5.731). 

Spore suspensions after extract pre-treatment were added to the 1:1 w:v sucrose solution to feed honeybees. In all three groups (control, spore suspensions after pre-treatment with *E. senticosus* in concentrations 0.4 mg/mL and in the concentration of 0.9 mg/mL), the sucrose solution was consumed at similar levels, i.e., observed daily consumption per one honeybee in the control was 0.0391 µL (±0.00950), in concentrations 0.4 mg/mL it was 0.0390 µL (± 0.01041) and in concentrations of 0.9 mg/mL it was 0.0413 µL (±0.01088). The level of nosemosis calculated using qPCR (quantitative polymerase chain reaction) was similar in all three groups (*p* > 0.5) and in the control it was equal to 250.9 × 10^4^ (±30.27) *N. ceranae* copies, in concentrations of 0.4 mg/mL it was 283.9 (±15.60) and in concentrations of 0.9 mg/mL it was 261.0 × 10^4^ (±46.2) *N. ceranae* copies. Honeybee mortality was similar (*p* > 0.5) in all three experimental groups. 

Summing up, there must be other mechanisms that cause administration of extracts to honeybees to lengthen honeybee lifespan and limit the development of nosemosis. We hypothesised that eleutherosides benefited the honeybee immune system. The effect of eleutherosides on honeybees should be checked in further studies to be conducted on pure substances.

### 2.3. Phytochemical Analysis of the Extracts and Honey

**Figure 5:**
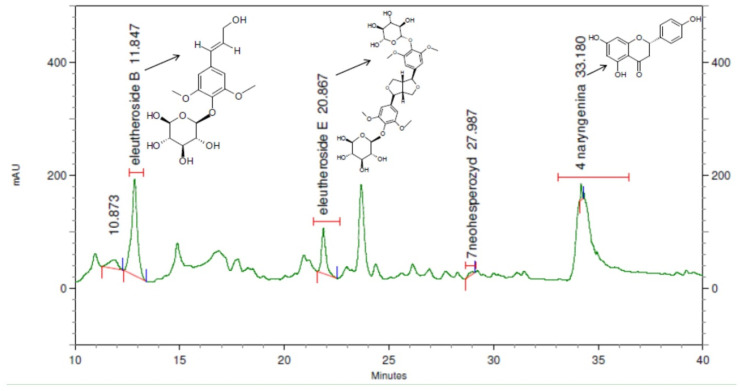
An exemplary chromatogram of the eleutheroside B, E and naringenin in the samples of freshly sourced extract.

The biological analysis showed that the 75% ethanol extract from the roots of *E. senticosus* had the optimum effect on the course of nosemosis. This extract was studied for its chemical constituents, stability during the 2-year storage and its residue in honey. Eleutherosides are major components present in the *E. senticosus* roots and, according to European Pharmacopoeia, the total amount for the sum of eleutheroside B and E should not be lower than 0.08%. In order to check which group of compounds may be responsible for an increase in the immune response in bees, all investigated extracts were subjected to HPLC-DAD (High-Performance Liquid Chromatography (HPLC) with a Diode-Array Detector) analysis to determine eleutherosides B and E and naringenin. The results obtained in this work showed eleutherosides B and E and naringenin to be present only in the roots of *E. senticosus* and *E. henryi*. The extract from the roots of *E. senticosus* contained eleutherosides B and E, 14.4 and 6.4 mg/g in dry extract respectively, and naringenin 0.17 mg/g in dry extract (Table 1). While, the roots of *E. henryi* contained eleutherosides B and E in the amount of 10.5 and 4.2 mg/g in dry extract, and respectively, naringenin 0.12 mg/g dry extract. Eleutheroside B was present in the majority of extracts, which may result from its higher polarity than eleutheroside E’s and naringenin’s. An exemplary chromatogram of eleutheroside B, E and naringenin in the extract is presented in Figure 5.

The next step was to determine the eleutheroside content after 2 years of the extract’s storage at 8 °C. There are no significant differences in eleutheroside B and E content between the freshly extracted dried roots and those after the 2-year storage (Supplementary Materials Table S4). Additionally, we did not detect eleutheroside B and E in honey made by honeybees whose diet was supplemented with *E. senticosus* extract (Supplementary Materials Figure S3).

## 3. Discussion

With the numbers of pollinators dramatically declining worldwide, the scientific community has been growing more and more concerned about the future of insects as fundamental elements of most terrestrial ecosystems. Therefore, we should look for preparations to support honeybee well-being. At present, many various types of synthetic substances are introduced into the environment, the accumulation of which may be harmful for insects. Thus, the search for a substance that would be beneficial for bees should be mainly based on natural substances. Furthermore, due to the fact that honeybee products are consumed by people, these substances must also be safe for people and their residues should not be present in honey or other bee products. Looking for substances that would increase bee resistance, we checked the effects of adaptogenic plants, which are defined as agents that support the ability to accommodate varying physical stresses, support health and prevent disease in both sick and healthy individuals through nonspecific effects and remain relatively safe and free of side effects. Adaptogenic plants selected for our study were: *Eleutherococcus senticosus, Garcinia cambogia, Panax ginseng, Ginkgo biloba, Schisandra chinensis* and *Camellia sinensis.* The *E. senticosus* root extract proved to be the most powerful. 

Plant-based metabolites cover a very broad range of polarities, which means that merely a part of the plant’s metabolites is present in the extract. Glycosides are well extracted with the 75% ethanol or methanol, sugars or phenolic acids may be extracted with water, and in turn, aglycons with non-polar solvents. The main group of the *Eleutherococcus senticosus* products on the market includes various extracts (ethanol, water). Cieśla et al. [42] studied the eleutherosides B and E content in the 75% ethanol extracts from the dried roots of *E. senticosus*. The extract contained 0.77 mg and 0.52 mg/g of the raw material dry weight, respectively [43]. According to Bączek et al. [44], the content of eleutheroside B and E in the dried roots of 4-year-old plants (methanol extract) was equal to 115.07 and 94.11 mg/100 g, respectively. In another Bączek’s study [45], the content of eleutherosides B and E in *E. senticosus* dried roots (methanol extract) depended on the plant’s age (2, 3, 4 years) and the month of samples’ collection (June, November). The results showed that the highest amount of eleutheroside B and E was produced in November by a 4-year-old plant and yielded in 103.57 and 105.14 mg/100 g, for eleutheroside B and E, respectively. This is in agreement with Bączek’s previous studies [46] and confirms a dependence of secondary metabolites’ production on the plant’s age, part and vegetation season in which plants were collected. The *E. senticosus* stem 50% ethanol extract can contain 10.72 mg/g extract of eleutheroside E [47]. Eleutherosides and naringenin are the main active compounds of *E. senticosus* extract and act as immunostimulants [48,49]; therefore, their content in the extract should be exactly determined. In our study, *E. senticosus* root 75% ethanol extract in concentration of 0.4 mg/mL proved most effective and responded to 5.76 µg/mL of eleutheroside B, 2.56 µg/mL of eleutheroside E and 0.068 µg/mL of naringenin. The optimum doses calculated per one honeybee/day were: c.a. 0.124 µg of eleutheroside B, 0.055 µg of eleutheroside E and 0.001 µg of naringenin. Nevertheless, the main therapeutic and prophylaxis effect against *Nosema* infection is probably connected only with eleutherosides as in other studies, naringenin from citrus fruit had a moderate effect on reducing *Nosema* spore loads and its main advantage was the extension of honeybees’ lifetime [50].

In choosing proper medicine, the minimum effective dose and stability of active compounds are very important issues. Therefore, when administering any supplements, one should be very careful because “the dose makes the poison”, as Paracelsus pointed out [51]. Honeybees are very fragile, and many phytochemicals showed toxic effects on their organisms, even in low concentrations, i.e., extracts from *Allium sativum* L., *Artemisia absinthium* L., *Laurus nobilis* L. [52,53,54] or clove oil and thymol [32]. Taking into account the weight of honeybees during our experiments, which was estimated at c.a. 128.4 mg, toxic doses calculated per one honeybee/day were: 0.216 µg of eleutheroside B, 0.096 µg of eleutheroside E and 0.003 µg of naringenin. Therefore, we recommend using *E. senticosus* extract in the concentration of 0.4 mg/mL for the field application. The 0.9 mg/mL concentration was also safe in apiary tests (Figure 4) but during the long time treatment, there were not significant differences between the effects of these two concentrations. Consequently, we recommend the lowest effective dose, which was 0.4 mg/mL *E. senticosus* extract containing 5.76 µg/mL of eleutheroside B, 2.56 µg/mL of eleutheroside E and 0.07 µg/mL of naringenin for the use in apiaries in order to prevent the risk of toxic effects on honeybees observed at higher *E. senticosus* extract concentrations, which might be caused by the uncontrolled usage or arbitrary increase in the frequency of administration by the beekeepers exceeding recommended doses.

In the next step, the stability of extracts during the 2-year storage was evaluated in this study. There were no significant differences between freshly sourced extracts and those after 2-year storage (Supplementary Materials Table S4.). Previous studies by Załuski and Janeczko [55] revealed that the long-time storage of the *Eleutherococcus* samples did not affect their chemical composition and there were no significant differences in polyphenols and minerals content. In cage tests, we also checked the properties of the 2-year storage extract on honeybees infected with nosemosis and there were no significant differences between the effects of freshly sourced extract and the 2-year stored one (data not presented).

Assessing the residue of eleutherosides in the final product, honey is the most important factor from the application and the consumer safety point of view. No pictures characteristic for eleutherosides were observed in the chromatogram on the basis of the HPLC-DAD analysis, which indicates a lack of eleutherosides in honey (Supplementary Materials Figure S3). It is worth noting that eleutherosides are safe for humans and could even be a desirable constituent of honey. Such a combination could strengthen its nutritional and pro-health properties. Administration of the standardised *E. senticosus* products is important for people who suffer from heart diseases and take digoxin-based drugs. Some European manufacturers offer standardised herbal extracts fortified with extracts containing higher eleutherosides concentration. The analysis of eleutherosides is highly effective in the assessment of the *E. senticosus*-based products and should be the first step to take when products are imported into the EU. 

Checking honeybees’ food preferences for proposed food supplements, it is very important to estimate the efficiency of proposed preparations in supporting honeybee well-being. In our study, honeybees willingly ate the sucrose solution with the addition of extracts from: *E. senticosus, G. cambogia, G. biloba* and *S. chinensis* (Figure 1). The extract from *P. ginseng* and *C. sinensis* caused the sucrose solution to be unattractive and honeybees were reluctant to take it (Table 1, Figure 1). Other studies also indicated that the effect of therapeutic agents was strongly correlated with honeybee food preferences and a low consumption of products was probably the reason for the lack of their declared healing effect, as was proven for Nosestat^®^, Phenyl salicylate and Vitafeed Gold^®^ [56]. Our study also indicated honeybee food preference as the main factor for the therapeutic agents’ healing effect. 

Insects are complex organisms with sophisticated social behaviour, feeding preferences and complex immunity. Tauber et al. [57] recommended that we should look for natural products to treat honeybees. However, it is not only the combination of results from survival and disease development as Tauber et al. [57] claimed, but also honeybee feeding preferences that should be considered during potential honeybee medicament tests.

Due to the fact that the *Nosema* spore cell wall is not the target for *E. senticosus* extract effect (Supplementary Figure S1), as was the case with porphyrins [58], there must be a different explanation of its healing effect. *E. senticosus* extract decreased the level of nosemosis, both after the infection as a curing treatment and used in the prophylaxis (Figure 1, Figure 2 and Figure 3, B: nosemosis treatment, C: nosemosis prevention). Hence, we hypothesise that *E. senticosus* extract effect is connected with its adaptogenic properties and provokes an adaptogenic effect in honeybees. The pharmacological assessment of adaptogens typically includes the evaluation of their stimulating, tonic and stress-protective effects. Here, we show for the first time such adaptogenic plant extracts’ impact on invertebrates in honeybees stressed by nosemosis.

Insects and other invertebrates lack certain characteristics of the adaptive immune system, instead they rely on innate immune responses to defend against foreign microorganisms and mount several defence reactions, including the activation of prophenoloxidase (proPO) into its active form phenoloxidase (PO) [59,60]. In our earlier studies [61], we observed the increased level of PO in the hemolymph of infected honeybee treated with *E. senticosus* extracts. The level of PO in honeybee hemolymph was checked using standard, previously described procedures [62,63,64]. This observed PO increment was strongly correlated with the *Nosema* infection. PO was at a physiologically low level in the hemolymph of non-infected honeybees treated with *E. senticosus* extracts. However, after the infection, the hemolymph of honeybees treated with *E. senticosus* extracts had much higher amounts of PO in comparison to honeybees untreated with *E. senticosus* extracts. This increase in PO level is very interesting due to the fact that insect PO in hemolymph is inhibited after the injection of fungal spores [65,66]. In our study [61], we observed that honeybees fed with *E. senticosus* extracts and infected with *Nosema* spp. spores had higher PO levels than those in control groups of *Nosema*-infected insects fed with the pure sucrose solution. Therefore, the effect of the *E. senticosus* extracts on honeybee had a similar adaptogenic response as on other animals, including humans [67,68]. We additionally hypothesised that these adaptogenic properties were connected with eleutherosides, the active compounds found exclusively in plants from the *Eleutherococcus* genus and undetected in other studied plant extracts. These active compounds are very stable in plant extracts and can be found in similar amounts even after two years from extraction (Supplementary Materials Table S4). In honeybees treated with such 2-year extracts, nosemosis level decreased comparably to the group of honeybees treated with freshly sourced extracts.

This adaptogenic effect of *E. senticosus* extract was also observed in our field studies (4.13.1. Field tests, Figure 4). Honeybee colonies which had been administered *E. senticosus* extract twice a year had lower winter debris and had lower levels of nosemosis than the colonies which were left untreated (Figure 4). Although there were natural, seasonal fluctuations in levels of nosemosis connected with its progress in honeybee colonies in temperate zones [17,69,70,71], colonies treated with *E. senticosus* extract always had lower *Nosema* load than colonies left untreated. These studies could be the basis for composing new functional honeybee food. Such food additives can strengthen honeybee colonies’ immunity and prevent colony losses. 

Natural products, such as herb extract, provide promising candidates for ‘bee medicines’ or for a safe prophylaxis [57]. *Eleutherococcus senticosus* extract is rich in many secondary metabolites with proven adaptogenic properties [67,72]. The pollinator fidelity hypothesis suggested that so-called “toxic nectar” is analogous to other floral structures that require specialisation of pollinators [24,73,74]. Honeybees taking such herb nectar might self-heal slightly or weakly infected colonies [15,17]. We bring out this hypothesis here because nectars rich in specific secondary compounds can decidedly benefit honeybees. *Eleutherococcus senticosus*, and its active compounds, eleutherosides, are considered the most powerful adaptogens, in the pool of all extracts that were selected for screening, for supporting immunity and improving resistance of honeybees.

## 4. Methods

### 4.1. Standards and Reagents

Ethanol was obtained from POCH (Polish Chemicals Reagents, Lublin, Poland). The standards of eleutheroside B ≥ 98.0% (HPLC), eleutheroside E ≥ 98.0% (HPLC) and naringenin ≥98.0% (HPLC), were obtained from Sigma-Adrich. LC-grade methanol (Methanol gradient grade for Liquid Chromatography) was purchased from J.T. Baker (Phillipsburg, NJ, USA). Ultrapure water was prepared using the Millipore Direct-Q3 purification system (Bedford, MA, USA). All other reagents were of analytical grade.

### 4.2. Plant Material

The roots and fruits of *E. senticosus* (Rupr. et Maxim.) Maxim. and *E. henryi* Oliv. were collected at the arboretum in Rogów (Poland) in October 2015 (voucher specimen numbers: ES01/2015, EH02/2015). All plant samples were deposited at the Department of Pharmaceutical Botany and Pharmacognosy, Nicolaus Copernicus University, Poland. The growth conditions were as follows: geographic data 51°49′ N and 19°53′ E, the average, long-term temperature −20.1 °C, the 6bth sub-climate (according to the United States Department of Agriculture Frost Hardiness Zones), and the second zone according to the Kórnik’s category [75]. The plants were grown on the acidic, luvic and sandy soils [75]. Plant materials’ identity was evaluated morphologically [76] and by HPLC-PDA analysis (see Section 4.6.), in comparison with reference substances (eleutherosides B and E).

### 4.3. Dried Material Extraction with 75% Ethanol

The air-dried roots and fruits (5 g each) were soaked in 50 mL 75% ethanol for 24 h. Next, the samples were subjected to triple UAE-type extraction (Ultrasonic-Assisted Extraction, ultrasonic bath, Polsonic, Warsaw, Poland) using 1 × 50 mL and 2 × 25 mL of 75% ethanol. The extraction was performed at room temperature for 15 min for each cycle. Finally, 100 mL of each extract was obtained. The solvents were dried with an evaporator under vacuum conditions at 45 °C and subjected to lyophilisation.

### 4.4. Dried Material Extraction with Chloroform

The roots and fruits were extracted using chloroform in the same way as the extraction with 75% ethanol.

### 4.5. Infusion Preparation

The infusion was prepared by adding 50 mL of distilled water (95 °C) to 5 g of the fruits or roots. The infusions were brewed for 15 min and were then filtered over Whatman No. 1 paper. The aqueous extracts were frozen and lyophilised.

### 4.6. Phytochemical Analysis of the Extracts and Honey

For this purpose, the Agilent 1200 Series HPLC system (Agilent Technologies, Santa Clara, CA, USA) was used equipped with a binary gradient solvent pump, a degasser and an autosampler. The separation of the analytes was carried out on the Zorbax Eclipse XDB-C18 column (4.6 × 150 mm, 5 µm particle size; Agilent Technologies, Santa Clara, CA, USA) maintained at 25 °C, using 3 µL injections and the flow rate was 1 mL/min. The solvents used were water containing 5% ACN (Acetonitrile solvent A) and water containing 60% ACN (solvent B). The following gradient elution program at the flow rate of 370 µL min^–1^ was applied: 0–5 min, 97% A–3% B; 6–30 min, 60% A–40% B; 31–45 min, 5% B–95% A; 45.1–60 min, 97% A–3% B. UV detection was conducted at 220 nm. Triplicate injections were made for each standard solution and sample. A stock solution (0.5 mg/0.5 mL) of each eleutherosides B and E and naringenin was prepared in 75% ethanol. Analytes’ content was determined from the corresponding calibration curves. The calibration functions of the eleutherosides B and E and naringenin were calculated using the peak area, concentration and mean values. There were three measurements for each concentration (0.0010, 0.0025, 0.010, 0.1 mg/mL). Linearity ranges for calibration curves were specified (1–100 µg/mL), and a correlation coefficient of 0.999 (R2), respectively. (Supplementary Materials Table S4).

### 4.7. Phytochemical Analysis of the Extracts: Stability of the Extracts in Time—HPLC-DAD Conditions of Eleutheroside B and E and Naringenin

The HPLC-DAD analysis was performed according to the method described in Section 4.6. A fresh extract and an extract after 2 years of storage at 8 °C were studied.

### 4.8. Phytochemical Analysis of the Honey: Eleutherococcus Extract Residues in Honey—HPLC-DAD of Eleutheroside B and E and Naringenin

The HPLC-DAD analysis was performed according to the method described in Section 4.6. Honey samples were collected from 15 beehives and extracted using the liquid–liquid extraction. In brief, 1 g of honey was dissolved in 20 mL of distilled water (left for 24 h, in a refrigerator). Next, the liquid–liquid extraction was performed using 5 × 20 mL of ethyl acetate. Ethyl acetate layers were evaporated, and the residues were used in HPLC-DAD analysis.

### 4.9. Animals, Cage and Field Tests

Honeybees, *Apis mellifera* L., were maintained with standard beekeeping management methods in the commercial apiary in Poland (50°30′ N 23°42 E) (Supplementary Figure S2). Although no permission is needed to administer experiments on insects, our research was planned in a way that reduced the number of honeybees to the minimum necessary for the proper conduction of these experiments. Honeybee samples left after conducting experiments are stored at the Department of Immunobiology UMCS (Maria Curie-Sklodowska University in Lublin, Poland) deposition.

### 4.10. Cage Tests: The Scheme of Administered Experiments

Cage tests were performed on *Apis mellifera* L. worker bees. After emerging, honeybees were kept under laboratory conditions (30 °C; Humidity = 65%) in wooden cages. In all experiments, honeybees were fed with a daily solution of 1:1 weight/volume sucrose: dH_2_O solution supplemented with extracts or/and other substances. The control honeybees were fed with a pure sucrose solution.

**Figure 6:**
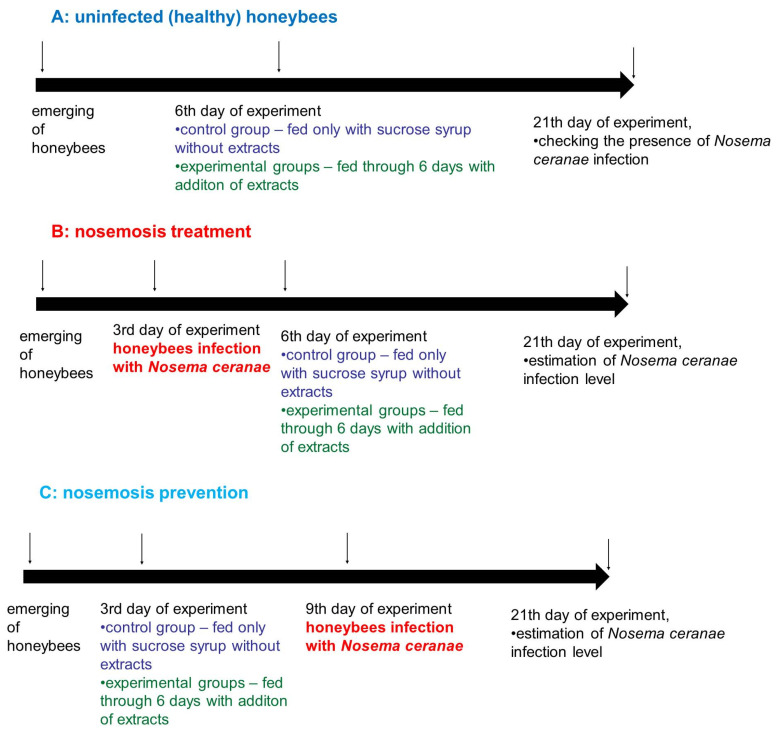
The scheme of administered experiments analysing the effect of extracts on honeybees which were uninfected and infected with *Nosema ceranae*.

Emerging honeybees were divided randomly into three treatments, “A”, “B” and “C” (Figure 6). Honeybees in treatment “A” (control, impact of extracts on uninfected honeybees’ lifespan), served as a control and were not infected with *N. ceranae*. In treatment “B” (impact of extracts on the treatment of nosemosis), to check whether the supplementation of honeybee diets with extracts influences the course of nosemosis, firstly, honeybees were *Nosema ceranae*-infected, and after that, fed with a sucrose syrup, containing extracts. In treatment “C” (impact of extracts on the prevention of nosemosis), to check whether the supplementation of honeybee diets with extracts protects honeybees against nosemosis, firstly, honeybees were fed with a sucrose syrup supplemented with extracts, and after that, were infected with *N. ceranae* spores.

To induce nosemosis, the honeybees were inoculated with a fresh solution containing 4 × 10^6^ *Nosema ceranae* spores/mL, in the amount of 8 µL per honeybee, and purified with classical methods according to the methodology described previously [77].

In each feeding group of cage tests, i.e., Section 4.10.1, Section 4.10.2 and Section 4.10.3, there were 3 cages settled by 40 honeybees in each (Table 3).

In all cage tests, dead bees were counted every day and the volume of eaten sucrose solution was estimated as the amount (mL) of food taken from the syringe. Additionally, at the end of the experiments, the level of *N*. *ceranae* infection was estimated.

#### 4.10.1. Cage Tests: Screening of Commercial Plant Extracts

Doses of the extracts, i.e., 0.2 and 1 mg of active compound in 1 mL of the sugar syrup were estimated on the basis of the manufacturer’s advice concerning an average daily dosage of these supplements, taking 130 mg as an average honeybee weight. The average weight of honeybees was established after weighing 50 randomly chosen specimens used in the experiments, and was estimated at 128.4 mg.

Feeding groups: Commercial extracts were bought in a local herbal store. Commercial extract samples are available in the Department of Immunobiology UMCS resources (Lublin, Poland; 51°14′44″N 22°32′26″E). Commercial extracts of *Eleutherococcus senticosus* (ES), *Garcinia gummi-gutta* (GG), *Panax ginseng* (PG), *Schisandra chinensis* (SC), *Camellia sinensis* (CS) and *Ginkgo biloba* (GB) were added to a sucrose syrup in two concentrations: 0.2 mg/mL (a) and 1 mg/mL (b), of declared concentration of an active compound. Uninfected and *Nosema*-infected honeybees from A, B and C variants (Figure 6) were divided into 14 feeding groups, i.e., (1) SS, (2) ESa, (3) ESb, (4) GGa, (5) GGb, (6) PGa, (7) PGb, (8) SCa, (9) SCb, (10) CSa, (11) CSb, (12) GBa, (13) GBb and (14) Fum, where (1) SS was 1:1 w:v 50% sucrose solution, and (14) Fum, where 25 mg bicyclohexylammonium fumagillin dissolved in 1 litre of sucrose solution, as in standard commercial use, was recommended.

#### 4.10.2. Cage Tests: Screening for the Best Method to Obtain the Eleutherococcus Extract

Laboratory extracts of *E. senticosus* (ES) and *E. henryi* (EH) were obtained using three different methods (water, chloroform and ethanol extraction) from these plants’ roots and fruits (Table 2).

Feeding groups: Uninfected and *Nosema*-infected honeybees from A, B and C variants were divided into 14 feeding groups, i.e., (1) SS, (2) ESrW, (3) ESrCh, (4) ESrEt, (5) ESfW, (6) ESfCh, (7) ESfEt, (8) EHrW, (9) EHrCh, (10) EHrEt, (11) EHfW, (12) EHfCh, (13) EHfEt and (14) Fum, where (1) SS was 1:1 w:v 50% sucrose syrup, and (14) Fum, where 25 mg bicyclohexylammonium fumagillin dissolved in 1 litre of the sucrose syrup, as in standard commercial use, was recommended. Pure laboratory extracts were added in the concentration of 0.4 mg/mL. Fumagilline was used as a positive control due to its proven antymicrosporidial/antynosemosis activity.

#### 4.10.3. Cage Tests: Screening for the Best Eleutherococcus Dose

*E. senticosus* ethanol root extract was added to the sucrose solution to the final concentrations of 0.05 mg/mL, 0.2 mg/mL, 0.4 mg/mL, 0.9 mg/mL and 1.5 mg/mL. Uninfected and *Nosema*-infected honeybees from A, B and C variants were divided into 7 feeding groups, i.e., (1) SS, (2) ES0.05, (3) ES0.2, (4) ES0.4, (5) ES0.9, (6) ES1.5 and (7) Fum, where (1) SS was 1:1 w:v sucrose syrup, and (7) Fum, where 25 mg bicyclohexylammonium fumagillin dissolved in 1 litre of the sucrose syrup, as in standard commercial use, was recommended.

### 4.11. Isolation of Total DNA from Honeybees and Molecular Detection of N. ceranae

Total DNA from uninfected and *N. ceranae*-infected *A. mellifera* was isolated using the DNeasy^®^ Plant Kit (Qiagen) according to the manufacturer’s instruction. To identify *N. ceranae* DNA in the investigated samples, duplex polymerase chain reaction (PCR) was conducted with primers: 321-APIS and 218-MITOC primers [76] in a 25 μL reaction mixture of the QIAGEN Taq PCR Core Kit (QIAGEN Inc.) containing 2.5 μL PCR buffer 5 μL Q solution, 0.1 mM dNTP (deoxynucleoside triphosphate) mixture, 0.7 U Taq DNA polymerase, 0.2 μM of each forward and reverse primers, approximately 0.15 μg of DNA template and ddH_2_O to a final reaction volume of 25 μL. For DNA amplification, the following PCR cycling conditions were used: 1 min at 94 °C, 1 min at 61.8 °C, and 1 min at 72 °C, repeated for 30 cycles, and 10 min at 72 °C.

### 4.12. Estimation of the Level of Nosemosis Level

Standard *Nosema* spp. spore counting: Samples were prepared from every group in two repeats to count *N. ceranae* spores. For one sample, 10 (cage tests) or 50 (field tests) honeybee abdomens were ground in 10 mL of sterile, distilled water and the number of *Nosema* spores was counted according to standard methods [77,78,79,80] using a haemocytometer and the Olympus BX61 light microscope. Furthermore, each sample was observed under bright field and differential interference contrast (DIC) to a proper differentiation of *N. ceranae* spores from other remains present in honeybees’ homogenates.

Real-time PCR for relative quantification *N. cearanae* was performed in accordance with the guidelines proposed in previous studies [77,80,81,82]. DNA was extracted from pooled whole abdomens of 5 bees (cage tests) or 50 bees (field tests). Samples were homogenised with liquid nitrogen, mortar and pestle. Pulverised abdomens were transferred to a microfuge tube, lysis solution was added and protocol run according to the manufacturer’s instructions for DNeasy^®^ Plant Kit (Qiagen). A qPCR analysis was performed with TOptical Gradient 96 (Biometra) using primer pairs specific for the 16SSU rDNA (ribosomal DNA) region for *N. ceranae* and *Apis mellifera* β-actin according to Coplay and Jabaji [18], Chen et al. [83] and Chen et al. [84], i.e., NceranaeF 5-CGGATAAAAGAGTCCGTTACC-3, NceranaeR 5-TGAGCAGGGTTCTAGGGAT-3, NcerProbe 5-⁄5HEX⁄CGTTACCCTTCGGGGAATCTTC⁄3IABkFQ⁄-3, ActinF 5-AGGAATGGAAGCTTGCGGTA-3, ActinR 5-AATTTTCATGGTGGATGGTGC-3, ActinProbe 5-56FAM⁄ATGCCAACACTGTCCTTTCTGGAGGTA⁄3IABkFQ⁄-3, resulting in respective amplified products of 250 and 181 bp. Each *N. ceranae* and *Apis mellifera* actin amplification mixture was prepared according to Coplay and Jabaji [18]. The nucleic acid of *N. cearanae* levels were quantified in all samples and each well in the PCR plate was loaded with 50 ng of DNA, all samples were run in triplicate technical runs. Standard curves and no template controls were run with each plate. Standard curve quantification was used to convert the resulting cycle threshold (C_T_) values to the number of copies of *N. ceranae* present in each sample. Serial dilutions using purified PCR products with known concentrations from 1 × 10^9^ to 1 × 10^2^ copies/μL were used to prepare the standard curve. Positive and no template controls were run with each plate. The products of the amplifications were separated on 2% agarose gel to exclude the presence of primer-dimer structures and non-specific products. The coefficient of correlation values was calculated for each sample to ensure the repeatability of amplicons. The copy number was expressed as the average *N. ceranae* copy number per bee.

### 4.13. Additional Tests

#### 4.13.1. Field Tests

Field tests were conducted in duplicate during two years in two commercial apiaries in Poland with the temperate continental climate, i.e., summers with average temperatures between 18 and 30 °C (i.e., 64.4 and 86.0 F) and winters, with average temperatures between −10 and 3 °C (i.e., 14.0 and 37.4 F). Each commercial apiary maintained more than 120 colonies, and for the purpose of the research, 30 colonies were chosen from each apiary. In the spring (March 2017), dead worker honeybees (winter debris) were sampled from hive bottom-drawers in order to select colonies for field tests. The worker honeybees were collected separately from each colony and examined for the presence of *Nosema* spp. spores. For this purpose, 50 worker-bees from each colony were collected and analysed by optical microscopy [79,80]. In this way, colonies were divided into healthy (non-infected) and infected with *Nosema* spp. Colonies in which *Nosema* spp. spores were found were put in a secluded place and no nosemosis treatment was administered. The health status of selected colonies was confirmed using PCR as a standard molecular method [76], and colonies infected solely by *Nosema ceranae* were chosen for the experiment. For the purpose of the experiment, 15 healthy colonies and 15 medium-infected colonies with the infestation level between 140.77 × 10^4^ (±24.86) and 166.4 × 10^4^ (±23.94) *Nosema* copy number/bee were selected. In each apiary, colonies were divided into 6 groups, i.e., (F1) 5 colonies as control, untreated group with healthy honeybees, (F2) 5 colonies with healthy honeybees fed with *E. senticosus* extract in the concentration of 0.4 mg/mL, (F3) 5 colonies with healthy honeybees fed with *E. senticosus* extract in the concentration of 0.9 mg/mL, (F4) 5 untreated colonies with *Nosema*-infected honeybees, (F5) 5 colonies with *Nosema*-infected honeybees fed with *E. senticosus* extract in the concentration of 0.4 mg/mL and (F6) 5 colonies with *Nosema*-infected honeybees fed with *E. senticosus* extract in the concentration of 0.9 mg/mL. The treated colonies were fed the sucrose solution with extracts four times, twice in spring (1–15 April 2017, 1–15 April 2018), and twice in late summer (1–15 September 2017, 1–15 September 2018). Dead bees from winter debris were collected from each studied colony during February of 2018 and 2019, weighted and the level of *Nosema* and type of infection were estimated. The level of *N. ceranae* infection in honeybee colonies was measured in September 2017, May 2018 and September 2018. For this purpose, at least 50 foragers at the sealed entrance of the hive were collected from each hive around noon [85]. Furthermore, first honey collected from each studied colony in May/June of 2017 and 2018 was checked for the presence of *E. senticosus* extract residues using HPLC-DAD.

#### 4.13.2. Impact of the Pre-Treatment of *Nosema ceranae* Spores with *Eleutherococcus* Extracts on the Spores’ Viability

The spore pre-treatment was performed similarly to previously described methods [58]. The intestines from 30 naturally *N. ceranae*-infected honeybees were isolated to obtain a fresh spore solution. The presence of the *N. ceranae* DNA in the solution was proven by DNA analysis. After isolation, the intestines were ground in phosphate-buffered saline (PBS), filtered through sterile mesh and washed twice with PBS. Next, spores’ suspension in the sucrose:water solution (1:1 w:v) was divided into three parts: (1) left untreated, (2) suspended in *E. senticosus* extract in the concentration of 0.4 mg/mL and (3) suspended in *E. senticosus* extract in the concentration of 0.9 mg/mL. These three spores’ suspensions were incubated on a rotary shaker (160 rpm) for 20 h at 25 °C. After the specified time, the spores were centrifuged for 15 min at 4000 rpm and washed extensively using sucrose:water solution (1:1 w:v). The procedure was repeated at least four times to remove any extract residues. All spore pre-treatments were carried out in parallel with controls, under exactly the same conditions using the fresh spore suspension without any extracts. The obtained spore solutions were divided into three portions. One mixed with sugar syrup was used to infect honeybees, the second was taken for the viability control and the third to the cell wall analysis using Scanning Electron Microscopy (SEM).

#### 4.13.3. *Nosema* Spores’ Viability Control

The spores’ viability control was performed according to previous studies [58] using SYTO 9 and propidium iodide. Quantifying spore viability was in accordance with Peng et al. [86], the spore samples were observed using the Axiovert 200M fluorescence microscope (Zeiss, Pliening, Germany).

#### 4.13.4. The *Nosema* Spore Cell Wall Analysis Under Scanning Electron Microscopy (SEM)

The SEM analysis (using VEGA LMU, TESCAN, s.r.o., Brno, Czech Republic) was performed similarly to previous studies [14]. A SEM portion of spores was fixed during 24 h in 5% gluteraldehyde (*v*/*v*) in 0.1 M phosphate buffer pH 7.3 for SEM. Then, the sample was washed in phosphate buffer prior to post-fixation in 1% osmium tetraoxide in 0.1 M-phosphate buffer for 24 h followed by washing in the same buffer. SEM samples were dehydrated by immersion for 15 min, each in fresh solutions of 30%, 50%, 75%, 90% and 100% acetone, and critical point-dried. The dried samples were mounted on specimen stubs using a double-sided adhesive tape and coated with gold. Coated samples were viewed under a VEGA LMU scanning microscope at 30 KV, measured and photographed.

### 4.14. Statistical Analyses

The analysis of variance (ANOVA) and simple correlations were carried out at the significance level of α = 0.05 using Statistica software (version 12.0, StatSoft Inc., Tulsa, OK, USA). Four types of tests were analysed: “Screening of commercial plant extracts”, “Screening for the best method to obtain the *Eleutherococcus* extract”, “Screening for the best *Eleutherococcus* dose”, and “Additional tests”. Tukey tests (one-way ANOVA, Statistica version 12.0, StatSoft Inc., Tulsa, OK, USA) at the significance level of α = 0.05 for all type of tests were used.

## 5. Conclusions and a Future Perspective

*Eleutherococcus senticosus* extract decreased the level of honeybee nosemosis, both after the infection as the curing treatment, and when used as prophylaxis. *E. senticosus* is a recognised plant-based adaptogen, so the mechanism of its effect on honeybees may be supposed to be similar to the one in other animals, including humans. The tested extracts did not affect *Nosema* spores’ viability or ability to infect honeybees. Therefore, there must be other mechanisms that cause administration of *E. senticosus* extracts to lengthen honeybee lifespan and limit the development of nosemosis. The hemolymph of *Nosema*-infected honeybees treated with *E. senticosus* extracts had much higher amounts of PO in comparison to honeybees untreated with *E. senticosus* extracts. We hypothesised that there is a synergism of all compounds present in the extract; nevertheless, the influence of eleutherosides (eleutheroside B + E) as the main extract ingredients on the honeybee immune system might be the most powerful, especially because adaptogens act in small doses, which was proven in this work as the minimum effective dose contained c.a. 5.76 µg of eleutheroside B, 2.56 µg of eleutheroside E and 0.07 µg of naringenin. The effects of eleutherosides and/or naringenin should be checked in further studies conducted on pure substances. In the future research, the administration of eleutherosides would thus be of interest. However, because of a medium solubility of eleutherosides in the aqueous solutions, the best option seems to be the use of the natural deep eutectic solvents (NADES) as non-toxic solvents. Presently, we know that sugar should be considered as a solvent, i.e., a syrup will extract compounds from plant material other than water. In particular, medium polar compounds like eleutherosides should be quite easily soluble in sugar-derived NADES. All these state-of-the-art research findings, from in vitro to nature, should be taken into account when drafting protocols for prospective honeybee medicament tests.

## 6. Highlights

Eleutherosides benefit honeybees. *E. senticosus* extracts had similar adaptogenic effects on honeybees as on other animals, including humans.

*E. senticosus* extract decreased the level of nosemosis, both after the infection—as the curing treatment, and when used as prophylaxis.

The minimum effective dose, i.e., 0.4 mg/mL of *E. senticosus* extract, contained 5.76 µg/mL of eleutheroside B, 2.56 µg/mL of eleutheroside E and 0.07 µg/mL of naringenin and proved to be optimal for honeybee nosemosis treatment and prophylaxis.

Honeybees fed with *E. senticosus* extracts and infected with *Nosema* spp. spores had higher PO levels than those in control groups of *Nosema*-infected insects fed with the pure sucrose solution.

Honeybee colonies which had been administered *E. senticosus* extract had lower winter debris and had lower nosemosis levels.

The active compounds’ concentration in *E. senticosus* extract is stable and effective for at least two years.

A combination of results from honeybee survival, disease development and feeding preferences should be considered while drafting prospective honeybee medicament tests.

## Figures and Tables

**Table 1:** Results of the cage tests: Screening of commercial plant extracts. The honeybee longevity: ++ the extract which was most efficient in the prolongation of the honeybee lifespan, + the extract prolonged the honeybee lifespan in comparison to the control group, - the extract reduced the honeybee lifespan in comparison to the control group. Reducing the level of nosemosis: ++ the extract which was most efficient in reducing the level of nosemosis, + the extract reduced the level of nosemosis in comparison to the control group, - the extract did not reduce the level of nosemosis in comparison to the control group. Food intake: + sucrose syrup with addition of the extract was consumed in a similar level as the pure sucrose syrup in the control group, - the extract was reluctantly consumed by honeybees in comparison to the control group.

	Honeybee Longevity	*Nosema* Infection	Food Intake
*Eleutherococcus senticosus* (ES)	++	++	+
*Garcinia gummi-gutta* (GG)	+	+	+
*Panax ginseng* (PG)	-	+	-
*Schisandra chinensis* (SC)	+	+	+
*Camellia sinensis* (CS)	-	-	-
*Ginkgo biloba* (GB)	+	-	+
Fumagillin (Fum)	-	++	+

**Table 2:** Designation of research groups under screening for the best method of obtaining the *Eleutherococcus* extract (acronyms are defined in Supplementary Materials Table S2).

The Type of Extraction Plant Species	Water	Chloroform	75% Ethanol
*E. senticosus* root	ESrW	ESrCh	ESrEt
*E. senticosus* fruit	ESfW	ESfCh	ESfEt
*E. henryi* root	EHrW	EHrCh	EHrEt
*E. henryi* fruit	EHfW	EHfCh	EHfEt

**Table 3:** The cage tests’ group sizes.

Section no.	Test	Feeding Groups	No. of cages in Each Feeding Group	No. of Bees in Each Cage	No. of Repeats	Total Honeybee Number in Each Experiment in One Treatment A, B or C
Section 4.10.1	Screening for commercial plant extracts.	14	3	40	2	3360
Section 4.10.2	Screening for the best method to obtain the *Eleutherococcus* extract	14	3	40	2	3360
Section 4.10.3	Screening for the best *Eleutherococcus* extract dose	7	6	40	2	3360

## Supplementary Materials

The following are available online: Table S1. Descriptions of acronyms defined in Section 4.10.1. Cage tests: Screening of commercial plant extracts. Table S2. Descriptions of acronyms defined in Section 4.10.2. Cage tests: Screening for the best method to obtain the *Eleutherococcus* extract. Table S3. Descriptions of acronyms defined in Section 4.10.3. Cage tests: Screening for the best *Eleutherococcus* dose. Table S4. Determination of eleutherosides B, E and naringenin in the biologically active extracts, freshly extracted and after 2-year storage (mg/g dry extract). Figure S1. Results of Additional tests. Figure S2. Exemplary images of cages from the cage tests and apiary with the experimental colonies. Figure S3. An exemplary chromatogram of the eleutheroside B and E in honey.

## References

[1] Aslan C.E., Liang C.T., Galindo B., Kimberly H., Topete W. (2016). The role of honey bees as pollinators in natural areas. Nat. Areas J..

[2] Prasad P.Y., Mackereth R.W., Hanley R.S., Qin W. (2015). Honey Bees (*Apis mellifera* L.) and Pollination Issues: Current status, impacts and potential drivers of decline. J. Agric. Sci..

[3] Zalasiewicz J., Waters C.N., Summerhayes C.P., Wolfe A.P., Barnosky A.D., Cearreta A., Crutzen P., Ellis E., Fairchild I.J., Gałuszka A. (2017). The Working Group on the Anthropocene: Summary of evidence and interim recommendations. Anthropocene.

[4] Crutzen P.J. (2002). Geology of mankind. Nature.

[5] Crutzen P.J., Stoermer E.F. (2000). The Anthropocene. Global Change Newsletter. Int. Geosph. Biosph. Programme.

[6] Ceballos G., Ehrlich P.R. (2018). The misunderstood sixth mass extinction. Science.

[7] Pimm S.L., Jenkins C.N., Abell R., Brooks T.M., Gittleman J.L., Joppa L.N., Raven P.H., Roberts C.M., Sexton J.O. (2014). The biodiversity of species and their rates of extinction, distribution, and protection. Science.

[8] Hallmann C.A., Sorg M., Jongejans E., Siepel H., Hofland N., Schwan H., Stenmans W., Müller A., Sumser H., Hörren T. (2017). More than 75 percent decline over 27 years in total flying insect biomass in protected areas. PLoS ONE.

[9] Sánchez-Bayo F., Wyckhuys K.A.G. (2019). Worldwide decline of the entomofauna: A review of its drivers. Biol. Conserv..

[10] Higes M., Martín R., Meana A. (2006). *Nosema ceranae*, a new microsporidian parasite in honeybees in Europe. J. Invertebr. Pathol..

[11] Fries I., Martín R., Meana A., García–Palencia P., Higes M. (2006). Natural infections of *Nosema ceranae* in European honey bees. J. Apic. Res..

[12] Chemurot M., Smet L., Brunain M., De Rycke R., de Graaf D.C. (2017). *Nosema neumanni* n. sp. (Microsporidia, Nosematidae), a new microsporidian parasite of honeybees, *Apis mellifera* in Uganda. Eur. J. Protistol..

[13] Panek J., Paris L., Roriz D., Mone A., Dubuffet A., Delbac F., Diogon M., El Alaoui H. (2018). Impact of the microsporidian *Nosema ceranae* on the gut epithelium renewal of the honeybee, *Apis Mellifera*. J. Invertebr. Pathol..

[14] Ptaszyńska A.A., Borsuk G., Mułenko W., Demetraki-Paleolog J. (2014). Differentiation of *Nosema apis* and *Nosema ceranae* spores under Scanning Electron Microscopy (SEM). J. Apicult. Res..

[15] Ptaszyńska A.A., Gancarz M., Hurd P.J., Borsuk G., Wiącek D., Nawrocka A., Strachecka A., Załuski D., Paleolog J. (2018). Changes in the bioelement content of summer and winter western honeybees (*Apis mellifera*) induced by *Nosema ceranae* infection. PLoS ONE.

[16] Huang Q., Evans J.D. (2020). Targeting the honey bee gut parasite *Nosema ceranae* with siRNA positively affects gut bacteria. BMC Microbiol..

[17] Ptaszyńska A.A., Paleolog J., Borsuk G. (2016). *Nosema ceranae* Infection Promotes Proliferation of Yeasts in Honey Bee Intestines. PLoS ONE.

[18] Copley T.R., Jabaji S.H. (2012). Honeybee glands as possible infection reservoirs of *Nosema ceranae* and *Nosema apis* in naturally infected forager bees. J. Appl. Microbiol..

[19] Wang D.I., Moeller F.E. (1971). Ultrastructural changes in the hypopharyngeal gland of worker honey bees infected by *Nosema apis*. J. Invert. Pathol..

[20] Ptaszyńska A.A., Borsuk G., Anusiewicz M., Mułenko W. (2012). Location of *Nosema* spp. spores within body of honey bee. Med. Weter..

[21] Lecocq A., Jensen A.B., Kryger P., Nieh J.C. (2016). Parasite infection accelerates age polyethism in young honey bees. Sci. Rep..

[22] Tofilski A., Kopel J. (1996). The influence of *Nosema apis* on maturation and flight activity of honey bee drones. Pszczel. Zesz. Nauk..

[23] Peng Y., Baer–Imhoof B., Millar A.H., Baer B. (2015). Consequences of *Nosema apis* infection for male honeybees and their fertility. Sci. Rep..

[24] Adler L.S. (2001). The ecological significance of toxic nectar. Oikos.

[25] Burnham A.J. (2019). Scientific Advances in Controlling *Nosema ceranae* (Microsporidia) Infections in Honey Bees (*Apis mellifera*). Front. Vet. Sci..

[26] Chen X., Wang S., Xu Y., Gong H., Wu Y., Chen Y., Zheng H. (2019). Protective potential of Chinese herbal extracts against microsporidian *Nosema ceranae*, an emergent pathogen of western honeybees, *Apis mellifera* L.. J. Asia Pac. Entomol..

[27] Kim J.H., Park J.K., Lee J.K. (2016). Evaluation of antimicrosporidian activity of plant extracts on *Nosema ceranae*. J. Apic. Sci..

[28] Giacomini J.J., Leslie J., Tarpy D., Palmer-Young E., Irwin R., Adler L.S. (2018). Medicinal value of sunflower pollen against bee pathogens. Sci. Rep..

[29] Bravo J., Carbonell V., Sepúlveda B., Delporte C., Valdovinos C.E., MartínHernández R., Higes M. (2017). Antifungal activity of the essential oil obtained from *Cryptocarya alba* against infection in honey bees by *Nosema ceranae*. J. Invertebr. Pathol..

[30] Arismendi N., Vargas M., López M.D., Barría Y., Zapata N. (2018). Promising antimicrobial activity against the honey bee parasite *Nosema ceranae* by methanolic extracts from Chilean native plants and propolis. J. Apic. Res..

[31] Ptaszyńska A.A., Borsuk G., Mułenko W., Wilk J. (2016). Impact of vertebrate probiotics on honeybee yeast microbiota and on the course of nosemosis. Med. Weter..

[32] Dumitru A., Chioveanu G., Ionita M., Dobre G., Mitrea I.L. (2017). “*In vitro*” studies on using natural essential oils in treatment of nosemosis in honeybees: Determination of the therapeutic Dose. Sci. Work. Vet. Medic. Ser. C.

[33] Mura A., Pusceddu M., Theodorou P., Angioni A., Floris I., Paxton R.J., Satta A. (2020). Propolis Consumption reduces *Nosema ceranae* infection of European honeybees (*Apis mellifera*). Insects.

[34] Palmer-Young E.C., Tozkar C.Ö., Schwarz R.S., Chen Y., Irwin R.E., Adler L.S., Evans J.D. (2017). Nectar and Pollen Phytochemicals Stimulate Honey Bee (Hymenoptera: Apidae) Immunity to Viral Infection. J. Econ. Entomol..

[35] Bernklau E., Bjostad L., Hogeboom A., Carlisle A.H.S.A. (2019). Dietary Phytochemicals, Honey Bee Longevity and Pathogen Tolerance. Insects.

[36] Porrini M.P., Garrido P.M., Gende L.B., Rossini C., Hermida L., Marcángeli J.A., Eguaras M.J. (2017). Oral administration of essential oils and main components: Study on honeybee survival and Nosema ceranae development. J. Apic. Res..

[37] Maistrello L., Lodesani M., Costa C., Leonardi F., Marani G., Caldon M., Mutinelli F., Granato A. (2008). Screening of natural compounds for the control of nosema disease in honeybees (*Apis mellifera*). Apidologie.

[38] Huang W.-F., Solter L.F., Yau P.M., Imai B.S. (2013). *Nosema ceranae* Escapes Fumagillin Control in Honey Bees. PLoS Pathog..

[39] Williams G.R., Sampson M.A., Shutler D., Rogers R.E. (2008). Does fumagillin control the recently detected invasive parasite *Nosema ceranae* in western honey bees (*Apis mellifera*)?. J. Invertebr. Pathol..

[40] van den Heever J.P., Thompson T.S., Curtis J.M., Ibrahim A., Pernal S.F. (2014). Fumagillin: An overview of recent scientific advances and their significance for apiculture. J. Agric. Food Chem..

[41] Dimpfel W., Schombert L., Keplinger–Dimpfel I.K., Panossian A. (2020). Effects of an adaptogenic extract on electrial activity of the brain in elderly subjects with mild cognitive impairment: A randomized, double–blind, placebo–controlled, two–armed cross–over study. Pharmaceuticals.

[42] Cieśla Ł., Waksmundzka-Hajnos M., Załuski D., Smolarz H., Hajnos M. (2011). HPTLC–densitometric method for determination of eleutherosides B, E and E1 in different *Eleutherococcus* species. J. Chromatogr. Sci..

[43] Nawrot-Hadzik I., Choromańska A., Abel R., Preissner R., Saczko J., Matkowski A., Hadzik J. (2020). Cytotoxic Effect of Vanicosides A and B from Reynoutria sachalinensis against Melanotic and Amelanotic Melanoma Cell Lines and in silico Evaluation for Inhibition of BRAFV600E and MEK1. Int. J. Mol. Sci..

[44] Bączek K. (2009). Accumulation of biologically active compounds in Eleuthero (Eleutherococcus senticosus /Rupr. et Maxim./ Maxim.) grown in Poland. Herba Pol..

[45] Bączek K., Przybył J.L., Kosakowska O., Węglarz Z. (2017). Accumulation of phenolics in eleuthero (*Eleutherococcus senticosus* (Rupr. t Maxim.) Maxim.) as affected by plant development. Acta Sci. Pol. Technol. Cultus..

[46] Bączek K. (2014). Diversity of *Eleutherococcus* genus in respect of biologically active compounds accumulation. Herba Pol..

[47] Ahn J., Um M.Y., Lee H., Jung C.H., Heo S.H., Ha T.Y. (2013). Eleutheroside E, an active component of *Eleutherococcus senticosus*, ameliorates insulin resistance in type 2 diabetic db/db mice. Evid. Complement. Altern. Med..

[48] Załuski D., Olech M., Kuźniewski R., Verpoorte R., Nowak R., Smolarz H.D. (2017). LC–ESI–MS/MS profiling of phenolics from *Eleutherococcus* spp. inflorescences, structure–activity relationship as antioxidants, inhibitors of hyaluronidase and acetylcholinesterase. Saudi Pharm. J..

[49] Venkateswara R.P., Kiran S.D.V.S., Rohini P., Bhagyasree P. (2017). Flavonoid: A review on Naringenin. J. Pharmacog. Phytochemistr..

[50] Borges D., Guzman-Novoa E. (2020). Goodwin PH (2020) Control of the microsporidian parasite *Nosema ceranae* in honeybees (*Apis mellifera*) using nutraceutical and immuno-stimulatory compounds. PLoS ONE.

[51] Grandjean P. (2016). Paracelsus Revisited: The Dose Concept in a Complex World. Basic. Clin. Pharmacol. Toxicol..

[52] Porrini M.P., Fernández N.J., Garrido P.M., Gende L.B., Medici S.K., Eguaras M.J. (2011). In vivo evaluation of antiparasitic activity of plant extracts on *Nosema ceranae* (Microsporidia). Apidologie.

[53] Pohorec K. (2004). Laboratory studies on the effect of standardized *Artemisia absinthium* L. extract on Nosema apis infection in the worker Apis mellifera. J. Apis. Sci..

[54] Damiani N., Fernández N.J., Porrini M.P., Gende L.B., Álvarez E., Buffa F., Brasesco C., Maggi M.D., Marcangeli J.A., Eguaras M.J. (2014). Laurel leaf extracts for honeybee pest and disease management: Antimicrobial, microsporicidal, and acaricidal activity. Parasitol. Res..

[55] Załuski D., Janeczko Z. (2015). Variation in phytochemicals and bioactivity of the fruits of *Eleutherococcus* species cultivated in Poland. Nat. Prod. Resear..

[56] Botías C., Martín–Hernández R., Meana A., Higes M. (2013). Screening alternative therapies to control Nosemosis type C in honey bee (*Apis mellifera iberiensis*) colonies. Res. Vet. Sci..

[57] Tauber J.P., Collins W.R., Schwarz R.S., Chen Y., Grubbs K., Huang Q., Lopez D., Peterson R., Evans J.D. (2019). Natural Product Medicines for Honey Bees: Perspective and Protocols. Insects.

[58] Ptaszyńska A.A., Trytek M., Borsuk G., Buczek K., Rybicka–Jasińska K., Gryko D. (2018). Porphyrins inactivate *Nosema spp. microsporidia*. Sci. Rep..

[59] Eleftherianos I., Revenis C. (2011). Role and Importance of Phenoloxidase in Insect Hemostasis. J. Innate Immun..

[60] Ferrandon D., Imler J.L., Hetru C., Hoffmann J.A. (2007). The *Drosophila* systemic immune response: Sensing and signalling during bacterial and fungal infections. Nat. Rev. Immunol..

[61] Ptaszyńska A.A., Cytryńska M., Mułenko W., Zdybicka–Barabas A., Borsuk G., Załuski D. (2019). Plant extracts for use in the treatment of honey bees nosemosis and to enhance bee resistance.

[62] Ptaszyńska A.A., Borsuk G., Zdybicka–Barabas A., Cytryńska M., Małek W. (2016). Are commercial probiotics and prebiotics effective in the treatment and prevention of honeybee nosemosis C?. Parasit. Res..

[63] Andrejko M., Zdybicka–Barabas A., Cytryńska M. (2014). Diverse effects of *Galleria mellonella* infection with entomopathogenic and clinical strains of Pseudomonas aeruginosa. J. Invertebr. Pathol..

[64] Zdybicka–Barabas A., Mak P., Jakubowicz T., Cytryńska M. (2014). Lysozyme and defense peptides as suppressors of phenoloxidase activity in *Galleria mellonella*. Arch. Insect Biochem. Physiol..

[65] Stączek S., Zdybicka–Barabas A., Pleszczyńska M., Wiater A., Cytryńska M. (2020). *Aspergillus* niger α–1,3–glucan acts as a virulence factor by inhibiting the insect phenoloxidase system. J. Invertebr. Pathol..

[66] Wojda I., Cytryńska M., Zdybicka–Barabas A., Kordaczuk J., Hoeger U., Harris J. (2020). Insect Defense Proteins and Peptides. Vertebrate and Invertebrate Respiratory Proteins, Lipoproteins and Other Body Fluid Proteins.

[67] Davydov M., Krikorian A.D. (2000). *Eleutherococcus senticosus* (Rupr. et Maxim.) Maxim. (Araliaceae) as an adaptogen: A closer look. J. Ethnopharmac..

[68] Asea A., Kaur P., Panossian A., Wikman K.G. (2013). Evaluation of molecular chaperons Hsp72 and neuropeptide Y as characteristic markers of adaptogenic activity of plant extracts. Phytomedicine.

[69] Traver B.E., Williams M.R., Fell R.D. (2012). Comparison of within hive sampling and seasonal activity of *Nosema ceranae* in honey bee colonies. J. Invertebr. Pathol..

[70] Mulholland G.E., Traver B.E., Johnson N.G., Fell R.D. (2012). Individual variability of *Nosema ceranae* infections in *Apis mellifera* colonies. Insects.

[71] Gisder S., Hedtke K., Möckel N., Frielitz M.-C., Linde A., Genersch E. (2010). Five–year cohort study of *Nosema* spp. in Germany: Does climate shape virulence and assertiveness of *Nosema ceranae*?. Appl. Environ. Microbiol..

[72] Adamczyk K., Olech M., Abramek J., Pietrzak W., Kuźniewski R., Bogucka-Kocka A., Nowak R., Ptaszyńska A.A., Rapacka-Gackowska A., Skalski T. (2019). *Eleutherococcus* Species Cultivated in Europe: A New Source of Compounds with Antiacetylcholinesterase, Antihyaluronidase, Anti-DPPH, and Cytotoxic Activities. Oxidative Med. Cell. Longev..

[73] Baker H.G., Baker I., Gilbert L.E., Raven P.H. (1975). Studies of nectar-constitution and pollinator–plant coevolution. Coevolution of plants and animals.

[74] Rhoades D.F., Bergdahl J.C. (1981). Adaptive significance of toxic nectar. Am. Nat..

[75] Tumiłowicz J., Banaszczak P. (2007). Trees and shrubs of Aquifoliaceae family in Rogów and Glinna arboreta. Rocz. Dendrol..

[76] eFloras Missouri Botanical Garden, St. Louis, MO & Harvard University Herbaria, Cambridge, MA. http://www.efloras.org.

[77] Fries I., Chauzat M.-P., Chen Y.-P., Doublet V., Genersch E., Gisder S., Higes M., Mcmahon D.P., Martínhernández R., Natsopoulou M., Dietemann V., Ellis J.D., Neumann P. (2013). Standard methods for nosema research. The COLOSS BEEBOOK: Volume II: Standard Methods for Apis Mellifera Pest and Pathogen Research.

[78] Martín–Hernández R., Meana A., Prieto L., Salvador A.M., Garrido-Bailón E., Higes M. (2007). Outcome of colonization of *Apis mellifera* by *Nosema ceranae*. Appl. Environ. Microbiol..

[79] Cantwell G.E. (1970). Standard methods for counting *Nosema* spores. Am. Bee J..

[80] Hornitzky M. (2008). Nosema Disease—Literature review and three surveys of beekeepers—Part 2.

[81] Bourgeois A.L., Rinderer T.E., Beaman L.D., Danka R.G. (2010). Genetic detection and quantification of *Nosema apis* and *N. ceranae* in the honey bee. J. Invertebr. Pathol..

[82] Evans J.D., Schwarz R., Chen Y., Budge G., Cornman R., Rúa P.D., Miranda J.D., Forêt S., Foster L., Gauthier L., Dietemann J.D., Ellis P.N. (2013). Standard methodologies for molecular research in *Apis mellifera*. The COLOSS BEEBOOK, Volume I: Standard Methods for Apis Mellifera Research V.

[83] Chen Y., Evans J., Zhou L., Boncristiani H., Kimura K., Xiao T., Litkowski A.M., Pettis J. (2009). Asymmetrical coexistence of *Nosema ceranae* and *Nosema apis* in honey bees. J. Invertebr. Pathol..

[84] Chen Y.P., Higgins J.A., Feldlaufer M.F. (2005). Quantitative real–time reverse transcription PCR analysis of deformed Wing virus infection in the honeybee (*Apis mellifera* L.). Appl. Environ. Microbiol..

[85] Meana A., Martín–Hernández R., Higes M. (2010). The reliability of spore counts to diagnose *Nosema ceranae* infections in honey bees. J. Apic. Res. Bee World.

[86] Peng Y., Lee–Pullen T.F., Heel K., Millar A.H., Baer B. (2014). Quantifying spore viability of the honey bee pathogen *Nosema apis* using flow cytometry. Cytom. Part A.

