# Supplementary material for: Extracts from Eleutherococcus senticosus (Rupr. et Maxim.) Maxim. Roots: A New Hope Against Honeybee Death Caused by Nosemosis
Source: Molecules. 2020 Sep 28;25(19):4452. doi: 10.3390/molecules25194452 (PMC7582972; doi:10.3390/molecules25194452)
Supplement: Supplementary file 1 [file molecules-25-04452-s001.pdf]

Supplementary materials Table S1. Descriptions of acronyms defined in section 4.10.1. Cage tests: Screening of commercial plant extracts (compare with Fig. 6. The scheme of administered experiments).

**Group abrrevation**	**Adaptogenic plant extracts or other additions**	**A**<br>**control, impact of extracts on uninfected honeybees**	**B**<br>**impact of extracts on the treatment of nosemosis**<br><br>***Nosema ceranae* infection on 3rd experimental day**	**C**<br>**impact of extracts on the prevention of nosemosis**<br><br>***Nosema ceranae* infection on 9th experimental day**
SS	Control, sucrose syrup without extracts	not infected honeybees<br>fed through all the experiment with a sucrose syrup without extracts	*N. ceranae*-infected honeybees<br>fed through all the experiment with a sucrose syrup without extracts	*N. ceranae*-infected honeybees<br>fed through all the experiment with a sucrose syrup without extracts
ES	*Eleutherococcus senticosus*	not infected honeybees<br>fed through 6 days with sucrose syrup supplemented with:<br>- 0.2 mg/mL of commercial extracts of *E. senticosus* (ESa),<br>-1 mg/mL of commercial extracts of *E. senticosus* (ESb).	honeybees were firstly *Nosema ceranae*-infected<br>and after that fed through 6 days with a sucrose syrup supplemented with:<br>- 0.2 mg/mL of commercial extracts of *E. senticosus* (ESa),<br>-1 mg/mL of commercial extracts of *E. senticosus* (ESb)	firstly honeybees were fed through 6 days with a sucrose syrup supplemented with:<br>- 0.2 mg/mL of commercial extracts of *E. senticosus* (ESa),<br>-1 mg/mL of commercial extracts of *E. senticosus* (ESb),<br>after that honeybees were infected with *N. ceranae*
GG	*Garcinia gummi-gutta*	not infected honeybees<br>fed through 6 days with sucrose syrup supplemented with:<br>- 0.2 mg/mL of commercial extracts of *G. gummi-gutta* (GGa),<br>- 1 mg/mL of commercial extracts of *G. gummi-gutta* (GGb).	honeybees were firstly *N. ceranae*-infected<br>and after that fed through 6 days with a sucrose syrup supplemented with:<br>- 0.2 mg/mL of commercial extracts of *G. gummi-gutta* (GGa),<br>- 1 mg/mL of commercial extracts of *G. gummi-gutta* (GGb).	firstly honeybees were fed through 6 days with a sucrose syrup supplemented with:<br>firstly honeybees were fed through 6 days with a sucrose syrup supplemented with:<br>- 0.2 mg/mL of commercial extracts of *G. gummi-gutta* (GGa),<br>- 1 mg/mL of commercial extracts of *G. gummi-gutta* (GGb),<br>after that honeybees were infected with *N. ceranae*
PG	*Panax ginseng*	not infected honeybees	honeybees were firstly *N. ceranae*-infected	firstly honeybees were fed through 6 days

		fed through 6 days with sucrose syrup supplemented with:<br>- 0.2 mg/mL of commercial extracts of *P. ginseng* (PGa),<br>- 1 mg/mL of commercial extracts of *P. ginseng* (PGb).	and after that fed through 6 days with a sucrose syrup supplemented with:<br>- 0.2 mg/mL of commercial extracts of *P. ginseng* (PGa),<br>- 1 mg/mL of commercial extracts of *P. ginseng* (PGb).	with a sucrose syrup supplemented with:<br>- 0.2 mg/mL of commercial extracts of *P. ginseng* (PGa),<br>- 1 mg/mL of commercial extracts of *P. ginseng* (PGb),<br>after that honeybees were infected with *N. ceranae*
SC	*Schisandra chinensis*	not infected honeybees<br>fed through 6 days with sucrose syrup supplemented with:<br>- 0.2 mg/mL of commercial extracts of *S. chinensis* (SCa),<br>- 1 mg/mL of commercial extracts of *S. chinensis* (SCb).	honeybees were firstly *N. ceranae*-infected and after that fed through 6 days with a sucrose syrup supplemented with:<br>- 0.2 mg/mL of commercial extracts of *S. chinensis* (SCa),<br>- 1 mg/mL of commercial extracts of *S. chinensis* (SCb).	firstly honeybees were fed through 6 days with a sucrose syrup supplemented with:<br>- 0.2 mg/mL of commercial extracts of *S. chinensis* (SCa),<br>- 1 mg/mL of commercial extracts of *S. chinensis* (SCb),<br>after that honeybees were infected with *N. ceranae*
CS	*Camellia sinensis*	not infected honeybees<br>fed through 6 days with sucrose syrup supplemented with:<br>-0.2 mg/mL of commercial extracts of *C. sinensis* (CSa),<br>- 1 mg/mL of commercial extracts of *C. sinensis* (CSb).	honeybees were firstly *N. ceranae*-infected and after that fed through 6 days with a sucrose syrup supplemented with:<br>-0.2 mg/mL of commercial extracts of *C. sinensis* (CSa),<br>- 1 mg/mL of commercial extracts of *C. sinensis* (CSb).	firstly honeybees were fed through 6 days with a sucrose syrup supplemented with:<br>-0.2 mg/mL of commercial extracts of *C. sinensis* (CSa),<br>- 1 mg/mL of commercial extracts of *C. sinensis* (CSb),<br>after that honeybees were infected with *N. ceranae*
GB	*Ginkgo biloba*	not infected honeybees<br>fed through 6 days with sucrose syrup supplemented with:<br>- 0.2 mg/mL of commercial extracts of *G. biloba* (GBa),<br>- 1 mg/mL of commercial extracts of *G. biloba* (GBb).	honeybees were firstly *N.ceranae*-infected and after that fed through 6 days with a sucrose syrup supplemented with:<br>- 0.2 mg/mL of commercial extracts of *G. biloba* (GBa),<br>- 1 mg/mL of commercial extracts of *G. biloba* (GBb).	firstly honeybees were fed through 6 days with a sucrose syrup supplemented with:<br>- 0.2 mg/mL of commercial extracts of *G. biloba* (GBa),<br>- 1 mg/mL of commercial extracts of *G. biloba* (GBb),<br>after that honeybees were infected with *N. ceranae*
Fum	Fumagillin	not infected honeybees<br>fed through 6 days with sucrose syrup supplemented with 25 mg	honeybees were firstly *N. ceranae*-infected and after that fed through 6 days with a sucrose syrup supplemented with 25 mg	firstly honeybees were fed through 6 days with a sucrose syrup supplemented with 25 mg bicyclohexylammonium fumagillin

		bicyclohexylammonium fumagillin dissolved in 1 litre of the sucrose solution.	bicyclohexylammonium fumagillin dissolved in 1 litre of the sucrose solution.	dissolved in 1 litre of the sucrose solution, after that honeybees were infected with *N. ceranae*

Colours of the A, B, and C groups are the same as in the corresponding Figure 1.

Supplementary materials Table S2. Descriptions of acronyms defined in section 4.10.2. Cage tests: Screening for the best method to obtain the *Eleutherococcus* extract (compare with Fig. 6. The scheme of administered experiments).

Group abrrevation	Adaptogenic plant extracts or other additions	A<br>control, impact of extracts on uninfected honeybees	B<br>impact of extracts on the treatment of nosemosis<br><br>*Nosema ceranae* infection on 3rd experimental day	C<br>impact of extracts on the prevention of nosemosis<br><br>*Nosema ceranae* infection on 9th experimental day
1. SS	Control, sucrose syrup without extracts	not infected honeybees<br>fed through all the experiment with a sucrose syrup without extracts	*N. ceranae*-infected honeybees<br>fed through all the experiment with a sucrose syrup without extracts	*N. ceranae*-infected honeybees<br>fed through all the experiment with a sucrose syrup without extracts
2. ESrW	*Eleutherococcus senticosus*	not infected honeybees<br>fed through 6 days with sucrose syrup supplemented with:<br>- 0.4 mg/mL of root water extract of *E. senticosus*	honeybees were firstly *Nosema ceranae*-infected<br>and after that fed through 6 days with a sucrose syrup supplemented with:<br>- 0.4 mg/mL of root water extract of *E. senticosus*	firstly honeybees were fed through 6 days with a sucrose syrup supplemented with:<br>- 0.4 mg/mL of root water extract of *E. senticosus*<br>after that honeybees were infected with *N. ceranae*
3. ESrCh		not infected honeybees<br>fed through 6 days with sucrose syrup supplemented with:<br>- 0.4 mg/mL of root chloroform extract of *E. senticosus*	honeybees were firstly *Nosema ceranae*-infected<br>and after that fed through 6 days with a sucrose syrup supplemented with:<br>- 0.4 mg/mL of root chloroform extract of *E. senticosus*	firstly honeybees were fed through 6 days with a sucrose syrup supplemented with:<br>- 0.4 mg/mL of root chloroform extract of *E. senticosus*<br>after that honeybees were infected with *N. ceranae*
4. ESrEt		not infected honeybees<br>fed through 6 days with sucrose syrup supplemented with:	honeybees were firstly *Nosema ceranae*-infected<br>and after that fed through 6 days with a	firstly honeybees were fed through 6 days with a sucrose syrup supplemented with:<br>- 0.4 mg/mL of root ethanl extract of *E.*

		- 0.4 mg/mL of root ethanl extract of *E. senticosus*	sucrose syrup supplemented with:<br>- 0.4 mg/mL of root ethanl extract of *E. senticosus*	*senticosus*<br>after that honeybees were infected with *N. ceranae*
5.ESfW		not infected honeybees<br>fed through 6 days with sucrose syrup supplemented with:<br>- 0.4 mg/mL of fruit water extract of *E. senticosus*	honeybees were firstly *Nosema ceranae*-infected<br>and after that fed through 6 days with a sucrose syrup supplemented with:<br>- 0.4 mg/mL of fruit water extract of *E. senticosus*	firstly honeybees were fed through 6 days with a sucrose syrup supplemented with:<br>- 0.4 mg/mL of fruit water extract of *E. senticosus*<br>after that honeybees were infected with *N. ceranae*
6. ESfCh		not infected honeybees<br>fed through 6 days with sucrose syrup supplemented with:<br>- 0.4 mg/mL of fruit chloroform extract of *E. senticosus*	honeybees were firstly *Nosema ceranae*-infected<br>and after that fed through 6 days with a sucrose syrup supplemented with:<br>- 0.4 mg/mL of fruit chloroform extract of *E. senticosus*	firstly honeybees were fed through 6 days with a sucrose syrup supplemented with:<br>- 0.4 mg/mL of fruit chloroform extract of *E. senticosus*<br>after that honeybees were infected with *N. ceranae*
7. ESfEt		not infected honeybees<br>fed through 6 days with sucrose syrup supplemented with:<br>- 0.4 mg/mL of fruit ethanol extract of *E. senticosus*	honeybees were firstly *Nosema ceranae*-infected<br>and after that fed through 6 days with a sucrose syrup supplemented with:<br>- 0.4 mg/mL of fruit ethanol extract of *E. senticosus*	firstly honeybees were fed through 6 days with a sucrose syrup supplemented with:<br>- 0.4 mg/mL of fruit ethanol extract of *E. senticosus*<br>after that honeybees were infected with *N. ceranae*
8.EHrW	*Eleutherococcus henryi*	not infected honeybees<br>fed through 6 days with sucrose syrup supplemented with:<br>- 0.4 mg/mL of root water extract of *E. henryi*	honeybees were firstly *Nosema ceranae*-infected<br>and after that fed through 6 days with a sucrose syrup supplemented with:<br>- 0.4 mg/mL of root water extract of *E. henryi*	firstly honeybees were fed through 6 days with a sucrose syrup supplemented with:<br>- 0.4 mg/mL of root water extract of *E. henryi*<br>after that honeybees were infected with *N. ceranae*
9.EHrCh		not infected honeybees<br>fed through 6 days with sucrose syrup supplemented with:<br>- 0.4 mg/mL of root chloroform extract of *E. henryi*	honeybees were firstly *Nosema ceranae*-infected<br>and after that fed through 6 days with a sucrose syrup supplemented with:<br>- 0.4 mg/mL of root chloroform extract of *E. henryi*	firstly honeybees were fed through 6 days with a sucrose syrup supplemented with:<br>- 0.4 mg/mL of root chloroform extract of *E. henryi*<br>after that honeybees were infected with *N. ceranae*

10.EHrEt		not infected honeybees<br>fed through 6 days with sucrose syrup supplemented with:<br>- 0.4 mg/mL of root ethanol extract of *E. henryi*	honeybees were firstly *Nosema ceranae*-infected<br>and after that fed through 6 days with a sucrose syrup supplemented with:<br>- 0.4 mg/mL of root ethanol extract of *E. henryi*	firstly honeybees were fed through 6 days with a sucrose syrup supplemented with:<br>- 0.4 mg/mL of root ethanol extract of *E. henryi*<br>after that honeybees were infected with *N. ceranae*
11.EHfW		not infected honeybees<br>fed through 6 days with sucrose syrup supplemented with:<br>- 0.4 mg/mL of root ethanol extract of *E. henryi*	honeybees were firstly *Nosema ceranae*-infected<br>and after that fed through 6 days with a sucrose syrup supplemented with:<br>- 0.4 mg/mL of root ethanol extract of *E. henryi*	firstly honeybees were fed through 6 days with a sucrose syrup supplemented with:<br>- 0.4 mg/mL of root ethanol extract of *E. henryi*<br>after that honeybees were infected with *N. ceranae*
12.EHfCh		not infected honeybees<br>fed through 6 days with sucrose syrup supplemented with:<br>- 0.4 mg/mL of fruit choroform extract of *E. henryi*	honeybees were firstly *Nosema ceranae*-infected<br>and after that fed through 6 days with a sucrose syrup supplemented with:<br>- 0.4 mg/mL of fruit choroform extract of *E. henryi*	firstly honeybees were fed through 6 days with a sucrose syrup supplemented with:<br>- 0.4 mg/mL of fruit choroform extract of *E. henryi*<br>after that honeybees were infected with *N. ceranae*
13.EHfEt		not infected honeybees<br>fed through 6 days with sucrose syrup supplemented with:<br>- 0.4 mg/mL of fruit ethanol extract of *E. henryi*	honeybees were firstly *Nosema ceranae*-infected<br>and after that fed through 6 days with a sucrose syrup supplemented with:<br>- 0.4 mg/mL of fruit ethanol extract of *E. henryi*	firstly honeybees were fed through 6 days with a sucrose syrup supplemented with:<br>- 0.4 mg/mL of fruit ethanol extract of *E. henryi*<br>after that honeybees were infected with *N. ceranae*
14.Fum	Fumagillin	not infected honeybees<br>fed through 6 days with sucrose syrup supplemented with 25 mg bicyclohexylammonium fumagillin dissolved in 1 litre of the sucrose solution.	honeybees were firstly *N. ceranae*-infected and after that fed through 6 days with a sucrose syrup supplemented with 25 mg bicyclohexylammonium fumagillin dissolved in 1 litre of the sucrose solution.	firstly honeybees were fed through 6 days with a sucrose syrup supplemented with 25 mg bicyclohexylammonium fumagillin dissolved in 1 litre of the sucrose solution, after that honeybees were infected with *N. ceranae*

Colours of the A, B, and C groups are the same as in the corresponding Figure 2.

Supplementary materials Table S3. Descriptions of acronyms defined in section 4.10.3. Cage tests: Screening for the best *Eleutherococcus* dose (compare with Fig. 6. The scheme of administered experiments).

Group abrrevation	Adaptogenic plant extracts or other additions	A<br>control, impact of extracts on uninfected honeybees	B<br>impact of extracts on the treatment of nosemosis<br><br>*Nosema ceranae* infection on 3rd experimental day	C<br>impact of extracts on the prevention of nosemosis<br><br>*Nosema ceranae* infection on 9th experimental day
1. SS	Control, sucrose syrup without extracts	not infected honeybees fed through all the experiment with a sucrose syrup without extracts	*N. ceranae*-infected honeybees fed through all the experiment with a sucrose syrup without extracts	*N. ceranae*-infected honeybees fed through all the experiment with a sucrose syrup without extracts
2. ES0.05	*Eleutherococcus senticosus* ethanol root extract	not infected honeybees fed through 6 days with sucrose syrup supplemented with:<br>- 0.05 mg/mL of *E. senticosus* extract	honeybees were firstly *Nosema ceranae*-infected and after that fed through 6 days with a sucrose syrup supplemented with:<br>- 0.05 mg/mL of *E. senticosus* extract	firstly honeybees were fed through 6 days with a sucrose syrup supplemented with:<br>- 0.05 mg/mL of *E. senticosus* extract<br>after that honeybees were infected with *N. ceranae*
3. ES0.2		not infected honeybees fed through 6 days with sucrose syrup supplemented with:<br>- 0.2 mg/mL of *E. senticosus* extract	honeybees were firstly *Nosema ceranae*-infected and after that fed through 6 days with a sucrose syrup supplemented with:<br>- 0.2 mg/mL of *E. senticosus* extract	firstly honeybees were fed through 6 days with a sucrose syrup supplemented with:<br>- 0.2 mg/mL of *E. senticosus* extract<br>after that honeybees were infected with *N. ceranae*
4. ES0.4		not infected honeybees fed through 6 days with sucrose syrup supplemented with:<br>- 0.4 mg/mL of *E. senticosus* extract	honeybees were firstly *Nosema ceranae*-infected and after that fed through 6 days with a sucrose syrup supplemented with:<br>- 0.4 mg/mL of *E. senticosus* extract	firstly honeybees were fed through 6 days with a sucrose syrup supplemented with:<br>- 0.4 mg/mL of *E. senticosus* extract<br>after that honeybees were infected with *N. ceranae*
5. ES0.9		not infected honeybees fed through 6 days with sucrose syrup supplemented with:	honeybees were firstly *Nosema ceranae*-infected and after that fed through 6 days with a	firstly honeybees were fed through 6 days with a sucrose syrup supplemented with:<br>- 0.9 mg/mL of *E. senticosus* extract

		- 0.9 mg/mL of *E. senticosus* extract	sucrose syrup supplemented with:<br>- 0.9 mg/mL of *E. senticosus* extract	after that honeybees were infected with *N. ceranae*
6. ES1.5		not infected honeybees<br>fed through 6 days with sucrose syrup supplemented with:<br>- 1.5 mg/mL of *E. senticosus* extract	honeybees were firstly *Nosema ceranae*-infected<br>and after that fed through 6 days with a sucrose syrup supplemented with:<br>- 1.5 mg/mL of *E. senticosus* extract	firstly honeybees were fed through 6 days with a sucrose syrup supplemented with:<br>- 1.5 mg/mL of *E. senticosus* extract<br>after that honeybees were infected with *N. ceranae*
7.Fum	Fumagillin	not infected honeybees<br>fed through 6 days with sucrose syrup supplemented with 25 mg bicyclohexylammonium fumagillin dissolved in 1 litre of the sucrose solution.	honeybees were firstly *N. ceranae*-infected<br>and after that fed through 6 days with a sucrose syrup supplemented with 25 mg bicyclohexylammonium fumagillin dissolved in 1 litre of the sucrose solution.	firstly honeybees were fed through 6 days with a sucrose syrup supplemented with 25 mg bicyclohexylammonium fumagillin dissolved in 1 litre of the sucrose solution,<br>after that honeybees were infected with *N. ceranae*

Colours of the A, B, and C groups are the same as in the corresponding Figure 3.

Supplementary materials Table S4. Determination of eleutherosides B, E, and naringenin in the biologically-active extracts, freshly-extracted and after 2-year storage (mg/g dry extract). Abbreviations: r^2 – correlation coefficient, t_R – retention time, n.s. – not studied.

					E. senticosus		*E. henryi*	
**Compound**	**Calibration curves**	r^2	Linear range (µg/mL)	t_R (min)	Freshly-extracted	2-year storage	Freshly-extracted	2-year storage
Eleutheroside B	y=23027120.358x-12370.245	0.999	1-100	11.847	14.4±0.1	13.1±0.3	10.5±0.19	n.s.
Eleutheroside E	y=18407229.351x-9132.121	0.999	1-100	20.867	6.4±0.2	5.9±0.05	4.2±0.6	n.s.
Naringenin	y=82000958.389x-12560.148	0.999	1-100	33.180	0.17±0.09	0.15±0.01	0.12±0.04	n.s.

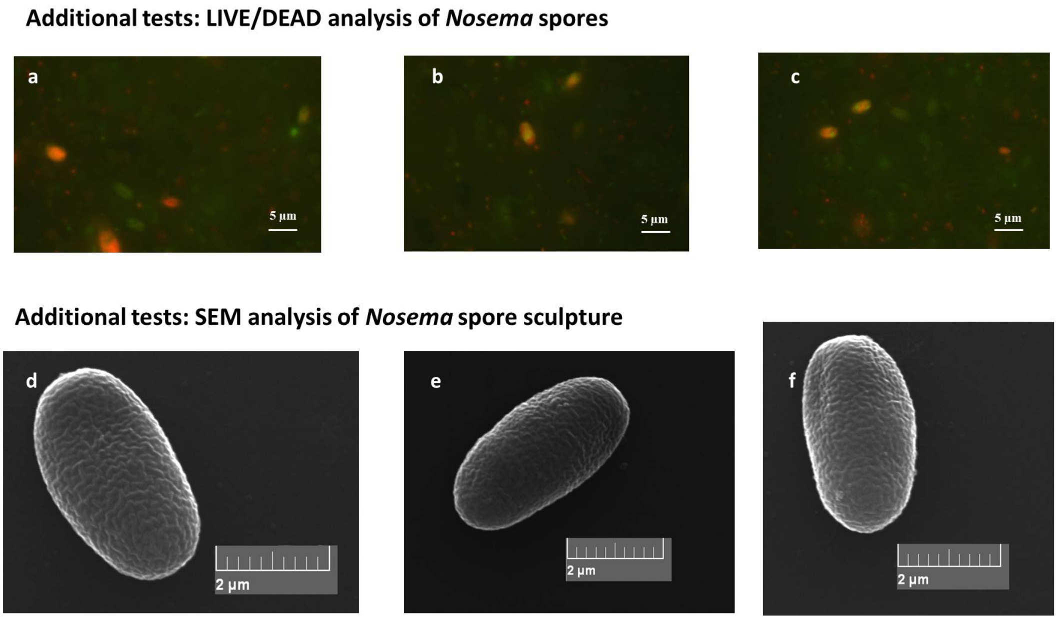

Figure S1. Results of Additional tests: (a-c) Exemplary images of the microsporidia spores dyed SYTO 9 and propidium iodide for LIVE/DEAD analysis showing no differences between extract-treated and untreated spores; (d-f) Exemplary images of the *Nosema* spore cell wall sculpture observed under Scanning Electron Microscopy (SEM).

Cage tests on honeybees

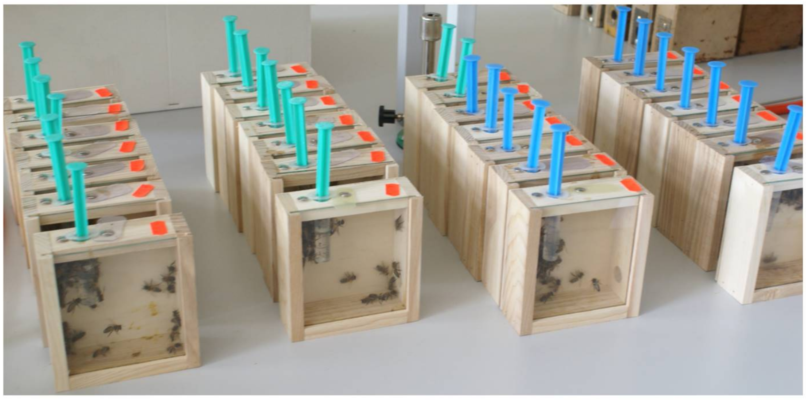

Field tests on honeybee colonies

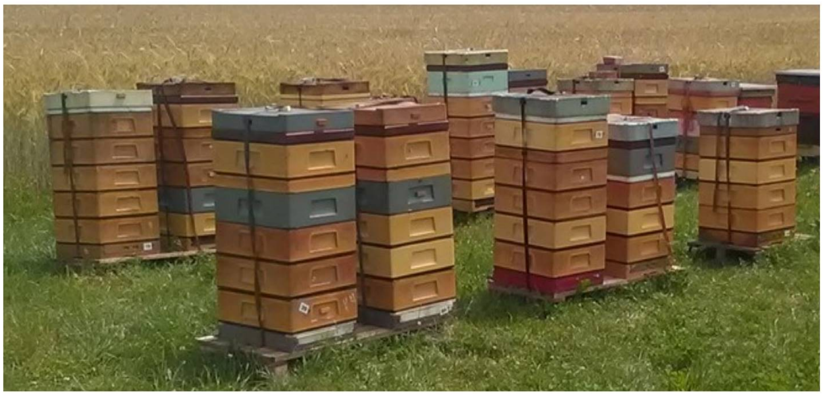

Figure S2. Exemplary images of cages from the cage tests and apiary with the experimental colonies.

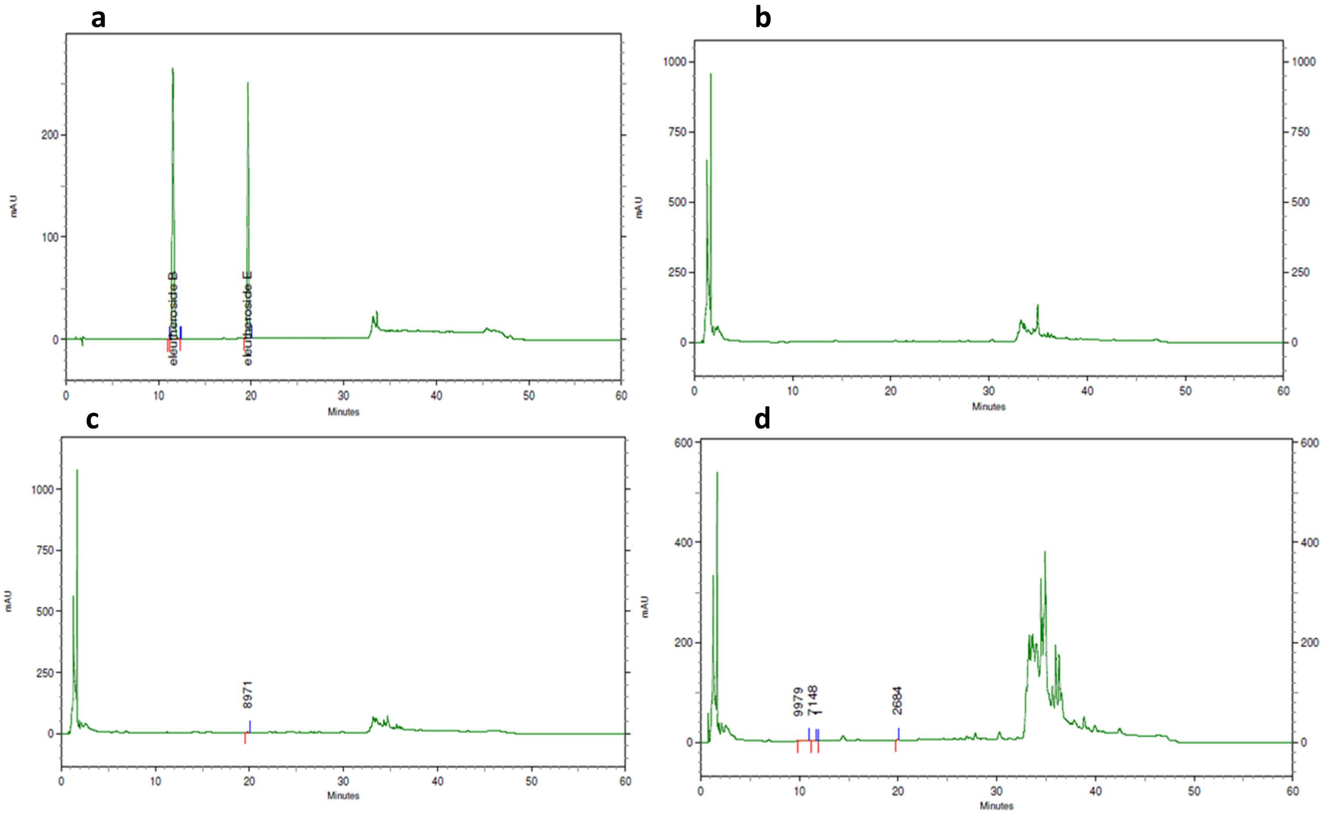

Figure S3. An exemplary chromatogram of the eleutheroside B and E in honey. a – standards of eleutheroside B and E (0.1 mg/mL), b-d – selected honey samples taken in May 2017 and June 2018, b- a honey sample taken in May of 2017 from apiary I, c- a honey sample taken in June 2018 from apiary I, d- a honey sample taken June 2018 from apiary II.
